# Evaluation of Adaptive Loop-Bandwidth Tracking Techniques in GNSS Receivers †

**DOI:** 10.3390/s21020502

**Published:** 2021-01-12

**Authors:** Iñigo Cortés, Johannes Rossouw van der Merwe, Jari Nurmi, Alexander Rügamer, Wolfgang Felber

**Affiliations:** 1 Satellite Based Positioning Systems Department, Fraunhofer IIS, Nordostpark 84, 90411 Nuremberg, Germany; Johannes.roussouw.vandermerwe@iis.fraunhofer.de (J.R.v.d.M.); alexander.ruegamer@iis.fraunhofer.de (A.R.); wolfgang.felber@iis.fraunhofer.de (W.F.); 2 Electrical Engineering, Tampere University, 33014 Tampere, Finland; jari.nurmi@tuni.fi

**Keywords:** global navigation satellite system (GNSS), adaptive scalar tracking loop (A-STL), fast adaptive bandwidth (FAB), fuzzy logic (FL), loop-bandwidth control algorithm (LBCA), piece-wise linear approximation of non-linearities (PLAN)

## Abstract

Global navigation satellite system (GNSS) receivers use tracking loops to lock onto GNSS signals. Fixed loop settings limit the tracking performance against noise, receiver dynamics, and the current scenario. Adaptive tracking loops adjust these settings to achieve optimal performance for a given scenario. This paper evaluates the performance and complexity of state-of-the-art adaptive scalar tracking techniques used in modern digital GNSS receivers. Ideally, a tracking channel should be adjusted to both noisy and dynamic environments for optimal performance, defined by tracking precision and loop robustness. The difference between the average tracking jitter of the discriminator’s output and the square-root Cramér-Rao bound (CRB) indicates the loops’ tracking capability. The ability to maintain lock characterizes the robustness in highly dynamic scenarios. From a system perspective, the average lock indicator is chosen as a metric to measure the performance in terms of precision, whereas the average number of visible satellites being tracked indicates the system’s robustness against dynamics. The average of these metrics’ product at different noise levels leads to a reliable system performance metric. Adaptive tracking techniques, such as the fast adaptive bandwidth (FAB), the fuzzy logic (FL), and the loop-bandwidth control algorithm (LBCA), facilitate a trade-off for optimal performance. These adaptive tracking techniques are implemented in an open software interface GNSS hardware receiver. All three methods steer a third-order adaptive phase locked loop (PLL) and are tested in simulated scenarios emulating static and high-dynamic vehicular conditions. The measured tracking performance, system performance, and time complexity of each algorithm present a detailed analysis of the adaptive techniques. The results show that the LBCA with a piece-wise linear approximation is above the other adaptive loop-bandwidth tracking techniques while preserving the best performance and lowest time complexity. This technique achieves superior static and dynamic system performance being 1.5 times more complex than the traditional tracking loop.

## 1. Introduction

A global navigation satellite system (GNSS) receiver needs to maintain lock on the satellite signals in order to decode their navigation message and determine the pseudorange [1]. Locking onto and following the signal’s Doppler frequency, carrier phase, and code phase is referred to as tracking [2]. A GNSS receiver must utilize the best possible means to track the signals since it leads to a better pseudo-range estimation, and in turn, to a more precise and reliable position, velocity, and time (PVT) solution. However, noise, receiver dynamics, and multi-path effects make this a challenging task.

Before tracking can be initiated, the signal must first be acquired: a coarse synchronization of the carrier and code is determined for each received GNSS signal [1]. The tracking uses this coarse estimation to initialize the scalar tracking loops (STLs). These loops improve the estimates by continuously monitoring the signal. They synchronize to the carrier frequency and Doppler offset with a frequency locked loop (FLL), the carrier phase with a phase locked loop (PLL), and the code phase with a delay locked loop (DLL). An STL tracks each of the received GNSS signals independently from the others. An alternative approach is to track and adapt the channels jointly with vector tracking (VT) [3], but currently there are several hardware implementation issues that currently limit practical use [4]. Therefore, VT is not considered for the remainder of the paper. The conventional digital tracking loop consists of a correlator, a discriminator, a loop filter, and a numerically controlled oscillator (NCO) [5]. The integration time $\tau_{\mathrm{int}}$, the correlator spacing, the discriminator type, the order, the noise bandwidth of the loop filter, and the oscillator determine the tracking performance at a given carrier-to-noise density ratio (*C*/*N*_0_).

Depending on the scenario, different loop settings are ideal. Dynamics stress the tracking loop’s accuracy, whereas noise limits precision [6,7]. The former is characterized by changes in position, velocity, acceleration, jerk, or even higher orders of dynamics. The latter arises due to the thermal noise, the oscillator phase noise, quantization noise, signal interference, and other noise sources [8]. Multi-path, fading effects, and scintillation also shape the noise and introduce biases [9]. The loop bandwidth of conventional STLs presents a trade-off between the ability to stay locked in the presence of dynamics and the amount of filtered noise to achieve better precision [6]. A large loop bandwidth is ideal for dynamic scenarios, as it presents tracking lock robustness, but results in low noise rejection and limits precision. A small loop bandwidth is suitable for noisy scenarios, as it rejects most noise, but dynamics can easily disrupt the loop and break the lock. Therefore, loop bandwidth tuning directly impacts tracking loop performance. However, it is impossible to choose a single, static value ideal for all scenarios and signals. Further, the geometry, path-loss, and environment between the receiver and each satellite in the constellation directly impact each signal’s dynamics and noise. Hence, even in a single scenario, each signal has an ideal loop bandwidth, which requires individualized tuning.

Adaptive loop-bandwidth tracking techniques address this problem by adapting the loop bandwidth of the STL depending on the scenario conditions [7]. These techniques determine a suitable loop bandwidth based on the tracking channel’s behavior. One method is the fast adaptive bandwidth (FAB) technique [10,11,12,13]. It uses a loop bandwidth dependent cost function, which serves to estimate the loop bandwidth in the next iteration. A second method is the fuzzy logic (FL) technique [14,15]: This technique updates the loop bandwidth based on a fuzzy decision of adequate inputs. The inputs can be any tracking channel information such as the discriminator’s output and its statistics. A third technique is the loop-bandwidth control algorithm (LBCA) [16]. This algorithm updates the loop bandwidth performing a weighted difference of estimated noise and estimated signal dynamics. Alternative tracking methods with Kalman filtering (KF) are promising and have gained popularity in the literature [17,18,19,20,21,22,23]; however, this approach still has some hardware implementation limitations and will not be considered for the remainder of the paper.

This paper expands a conference paper [7] by including the theory of adaptive tracking techniques and improving the scope of the results. It implements various adaptive loop-bandwidth tracking techniques on a GNSS receiver and verifies them. Furthermore, it presents a method to evaluate the performance and complexity of these techniques. The FAB, the FL, and the LBCA are implemented in the GOOSE© receiver [24,25]. The GOOSE receiver is a GNSS receiver with an open software interface. This receiver is tested in simulated scenarios with different dynamics and noise cases. This paper aims to achieve a static and dynamic system performance comparison between different adaptive tracking techniques. The system performance is also compared with the complexity of each implemented algorithm. It leads to a complete evaluation of the implemented adaptive tracking techniques.

The rest of the paper is organized as follows. Section 2 presents a detailed STL background, followed by a precise description of the implemented variable loop-bandwidth tracking techniques in Section 3. Section 4 analyzes the metric used to evaluate the tracking and system performance and evaluates the complexity of adaptive loop-bandwidth tracking techniques. The experimental setup and implementation in an open software interface GNSS hardware receiver are described in Section 5. Section 6 presents the results of the adaptive loops. These results are synthesized and discussed in Section 7. Finally, Section 8 concludes and indicates future work.

## 2. Scalar Tracking Loops

This section presents an introduction to standard digital STLs. Two models explain the operation of the STL. First, the non-linear model in the discrete-time domain describes the main components of an STL. Second, a linear model in the z-domain addresses the STL transfer function and the STL discrete state space model (SSM). These two representations include sufficient background to describe the behavior of STLs fully.

### 2.1. Non-Linear Model

**Figure 1:**
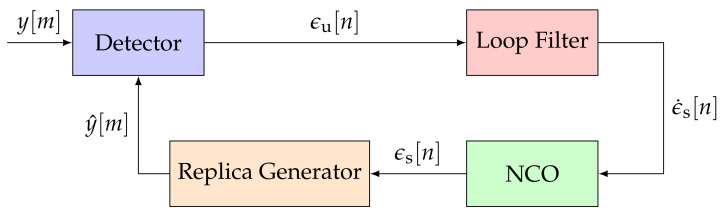
Architecture of a conventional scalar tracking loop (STL). ©IEEE. Adapted, with permission, from [7].

GNSS receivers commonly deploy FLLs, PLLs, and DLLs [1]. All three of these loops track a different signal parameter (i.e., Doppler frequency, carrier phase, or code phase) but have similar architectures. Figure 1 shows the non-linear STL model in the discrete-time domain [2]. The STL consists of a detector, a loop filter, an NCO, and a replica generator.

**Figure 2:**
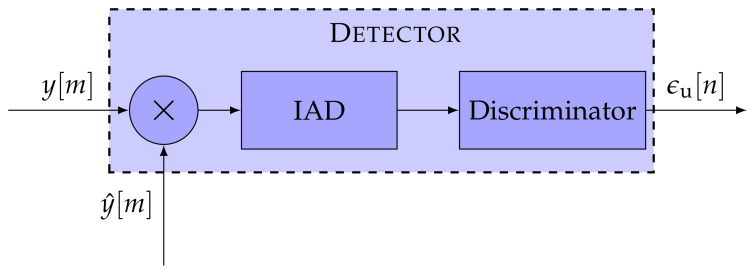
Architecture of the detector. ©IEEE. Adapted, with permission, from [7].

The first STL component is the detector. The detector calculates the error $\epsilon_{u}\left[n\right]$ of the representative tracking parameter. The error $\epsilon_{u}\left[n\right]$ is determined by comparing the incoming signal y[m] and an estimated replica of the signal $\hat{y}\left[m\right]$. Figure 2 presents the general structure of a detector. A GNSS receiver correlates an input signal with a locally generated replica to retrieve the GNSS signal. Therefore, a multiplier and an integration and dump (IAD) stage correlate a vector on input data samples, to extract the correlation in-phase and quadrature-phase (IQ) samples. The correlator decimates the signal to a sample-rate related to the integration time $\tau_{\mathrm{int}}$, resulting in multi-rate processing. The noise (i.e., the variance of the error $\epsilon_{u}\left[n\right]$) is inversely proportional to the integration time $\tau_{\mathrm{int}}$ of the IAD. Hence, the larger the integration time $\tau_{\mathrm{int}}$, the higher the processing gain—the less noisy the errors $\epsilon_{u}\left[n\right]$. However, with larger integration time, the loop response becomes slower, and consequently, it is less sensitive against signal dynamics. The last component of the detector is the discriminator function. It determines the error $\epsilon_{u}\left[n\right]$ for a specific signal parameter (i.e., Doppler frequency, carrier phase, or code phase) based on the correlation IQ samples. Each tracked signal parameter requires a different discriminator.

The second component of the STL is the loop filter. It shapes the noisy errors $\epsilon_{n}\left[n\right]$ to a smoothed error rate $\dot{\epsilon }\left[n\right]$. Hence, the filter input is an un-smoothed signal, and the filter output a smoothed one. The loop bandwidth affects the response time and noise suppression capabilities of the loop filter. Small loop bandwidths are ideal for suppressing unwanted noise, whereas large loop bandwidths respond quicker to signal dynamics. The loop filter is typically implemented as an infinite impulse response (IIR) filter [1,2,5]. The order of the IIR loop filter regulates the robustness of the tracking against high-order dynamics. A higher-order loop filter can track higher-order components of the error signal but adds complexity, and the possibility for instability in the system. The resulting smoothed error rate ${\dot{\epsilon }}_{s}\left[n\right]$ drives an NCO. The NCO integrates the output of the loop filter and sends the smoothed error $\epsilon_{s}\left[n\right]$ to the replica generator. The replica generator reconstructs a new estimate $\hat{y}\left[m\right]$ for the for the signal. The tracking loop minimizes the error $\epsilon_{u}\left[n\right]$ such that the generated replica signal $\hat{y}\left[m\right]$ converges to the input signal y[m].

### 2.2. Linear Model

**Figure 3:**
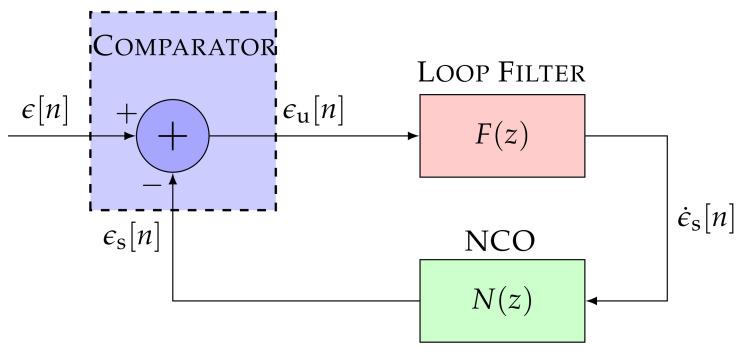
Linear model of the STL. ©IEEE. Adapted, with permission, from [7].

The linear model of an STL simplifies Figure 1 by removing the non-linear mapping of the discriminator and the multi-rate conversion of the correlator [2]. A discrete approximation of continuous-time systems is performed. This section considers the backward Euler transform (BET) [26,27] as it is used in current STL implementations. Figure 3 presents the linear model in the z-domain. The input signal ϵ[n] represents the carrier phase offset, the frequency Doppler, or the code phase offset of the input signal y[m]. The same functional blocks to Figure 1 are visible, but linearized.

The STL transfer function is often analyzed without considering the SSM representation analysis. This hinders the comparison with other tracking methods that are commonly represented by its SSM representation (e.g., KF-based STLs). Therefore, it is necessary to consider the transfer function and SSM representation since they are valuable concepts that serve to understand the STL behavior fully. From Figure 3, the controllable canonical form of the discrete SSM can be calculated [28]:

(1) $$\begin{array}{c} \frac{x_{p\times 1}\left[n\right]-x_{p\times 1}\left[n-1\right]}{\tau_{\mathrm{int}}}=\underset{A_{{\mathrm{cont}}_{p\times p}}}{\underset{︸}{\left[\begin{array}{ccccc} 0 & 1 & 0 & \cdots & 0\\ 0 & 0 & 1 & \cdots & 0\\ \vdots & \vdots & \vdots & \ddots & \vdots \\ 0 & 0 & 0 & \cdots & 1\\ 0 & 0 & 0 & \cdots & 0 \end{array}\right]}}x_{p\times 1}\left[n\right]+\underset{B_{{\mathrm{cont}}_{p\times 1}}}{\underset{︸}{\left[\begin{array}{c} \alpha_{p-1}\\ \alpha_{p-2}\\ \vdots \\ \alpha_{0} \end{array}\right]}}\epsilon_{u}\left[n\right] \end{array}$$

(2) $$\begin{array}{c} \epsilon_{s}\left[n\right]=\underset{C_{{\mathrm{cont}}_{1\times p}}}{\underset{︸}{\left[\begin{array}{ccccc} 1 & 0 & 0 & \cdots & 0 \end{array}\right]}}x_{p\times 1}\left[n\right]=x_{1}\left[n\right] \end{array}$$

where x is the discrete state vector, *p* is the order of the STL, $A_{\mathrm{cont}}$ is the state matrix, $B_{\mathrm{cont}}$ is the input matrix, and $C_{\mathrm{cont}}$ is the output matrix of the SSM in the continuous-time domain. $B_{\mathrm{cont}}$ contains the coefficients $\alpha_{k}$ of the STL. Equation (1) can be further developed:

(3) $$\begin{array}{c} x_{px1}\left[n\right]=\underset{A_{{\mathrm{discr}}_{p\times p}}}{\underset{︸}{{\left(I_{p\times p}-A_{{\mathrm{cont}}_{p\times p}}\tau_{\mathrm{int}}\right)}^{-1}}}x_{p\times 1}\left[n-1\right]+\underset{B_{{\mathrm{discr}}_{p\times 1}}}{\underset{︸}{{\left(I_{p\times p}-A_{{\mathrm{cont}}_{p\times p}}\tau_{\mathrm{int}}\right)}^{-1}B_{{\mathrm{cont}}_{p\times 1}}\tau_{\mathrm{int}}}}\epsilon_{u}\left[n\right] \end{array}$$

where $I_{p\times p}$ is the identity matrix, $A_{\mathrm{discr}}$ the discrete state matrix and $B_{\mathrm{discr}}$ the discrete control matrix.

Extending the previous equation, the following expression is achieved:

(4) $$\begin{array}{c} x_{p\times 1}\left[n\right]=\left[\begin{array}{ccccc} 1 & \tau_{\mathrm{int}} & \tau_{\mathrm{int}}^{2} & \cdots & \tau_{\mathrm{int}}^{p-1}\\ 0 & 1 & \tau_{\mathrm{int}} & \cdots & \tau_{\mathrm{int}}^{p-2}\\ \vdots & \vdots & \ddots & \ddots & \vdots \\ 0 & 0 & 0 & \cdots & \tau_{\mathrm{int}}\\ 0 & 0 & 0 & \cdots & 1 \end{array}\right]x_{p\times 1}\left[n-1\right]+\left[\begin{array}{c} \sum_{k=0}^{p-1}\alpha_{k}\tau_{\mathrm{int}}^{p-k}\\ \sum_{k=0}^{p-1}\alpha_{k}\tau_{\mathrm{int}}^{p-k-1}\\ \vdots \\ \alpha_{0}\tau_{\mathrm{int}} \end{array}\right]\epsilon_{u}\left[n\right] \end{array}$$

Performing the z-transform Z(·) of Equations (2) and (4), the open loop transfer function $H_{o}\left(z\right)$ of the discrete SSM can be obtained:

(5) Ho(z)=Z(ϵs)Z(ϵu)=Ccont1×pIp×p−Adiscrp×pz−1−1Bdiscrp×1==∑k=0p−1αkτintp−k(1−z−1)p−k=∑k=0p−1αkτintp−k−1(1−z−1)p−k−1︸F(z)τint1−z−1∑k=0p−1αkτintp−k−1(1−z−1)p−k−1︸N(z)

where F(z) is the transfer function of the loop filter and N(z) the transfer function of the NCO.

From the open loop transfer function, the closed loop transfer function $H_{c}\left(z\right)$ can be obtained:

(6) $$\begin{array}{c} H_{c}\left(z\right)=\frac{Z(\epsilon_{s})}{Z(\epsilon )}=\frac{H_{o}\left(z\right)}{1+H_{o}\left(z\right)}=\frac{F(z)\cdot N(z)}{1+F(z)\cdot N(z)} \end{array}$$

The zeros and poles of the closed loop transfer function depend on the coefficients of the tracking loop $\alpha_{k}$ and the integration time $\tau_{\mathrm{int}}$. Hence, the one-sided normalized bandwidth $B_{N}$ of the closed-loop transfer function is dependant on these coefficients:

(7) $$\begin{array}{c} 2B_{N}\left(\alpha_{0},\alpha_{1},\cdots ,\alpha_{p-1}\right)=2B\left(\alpha_{0},\alpha_{1},\cdots ,\alpha_{p-1}\right)\tau_{\mathrm{int}}=\frac{1}{2\pi j}\oint_{|z|=1}H_{c}\left(z\right)H_{c}\left(z^{-1}\right)z^{-1}dz \end{array}$$

where *B* is the one-sided equivalent noise bandwidth.

**Figure 4:**
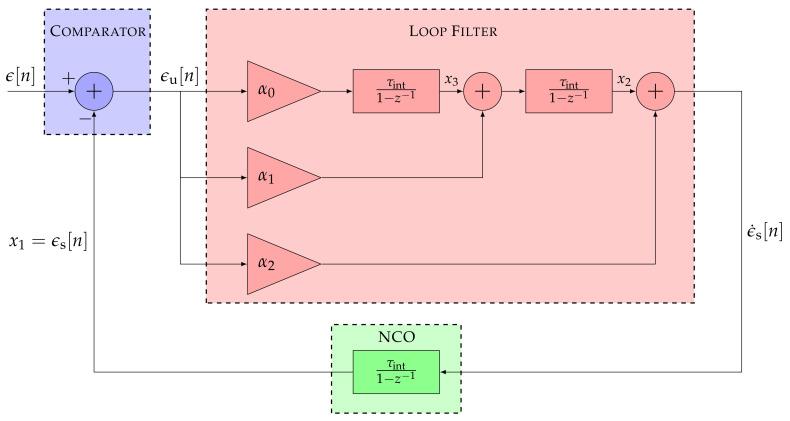
Linear model of a third-order STL.

The adaptive tracking techniques are implemented in a third-order STL. Therefore, the SSM and transfer function of a third-order STL are analyzed in Figure 4 displaying its linear model.

The equivalent discrete SSM is:

(8) $$\begin{array}{c} \left[\begin{array}{c} x_{1}\left[n\right]\\ x_{2}\left[n\right]\\ x_{3}\left[n\right] \end{array}\right]=\left[\begin{array}{ccc} 1 & \tau_{\mathrm{int}} & \tau_{\mathrm{int}}^{2}\\ 0 & 1 & \tau_{\mathrm{int}}\\ 0 & 0 & 1 \end{array}\right]\times \left[\begin{array}{c} x_{1}\left[n-1\right]\\ x_{2}\left[n-1\right]\\ x_{3}\left[n-1\right] \end{array}\right]+\left[\begin{array}{c} \alpha_{2}\tau_{\mathrm{int}}+\alpha_{1}\tau_{\mathrm{int}}^{2}+\alpha_{0}\tau_{\mathrm{int}}^{3}\\ \alpha_{1}\tau_{\mathrm{int}}+\alpha_{0}\tau_{\mathrm{int}}^{2}\\ \alpha_{0}\tau_{\mathrm{int}} \end{array}\right]\epsilon_{u}\left[n\right] \end{array}$$

(9) $$\begin{array}{c} \epsilon_{s}\left[n\right]=\left[\begin{array}{ccc} 1 & 0 & 0 \end{array}\right]\times \left[\begin{array}{c} x_{1}\left[n\right]\\ x_{2}\left[n\right]\\ x_{3}\left[n\right] \end{array}\right]=x_{1}\left[n\right] \end{array}$$

The open loop transfer function is characterized as:

(10) $$\begin{array}{c} H_{o_{3}}\left(z\right)=\frac{\alpha_{2}\tau_{\mathrm{int}}{\left(1-z^{-1}\right)}^{2}+\alpha_{1}\tau_{\mathrm{int}}^{2}\left(1-z^{-1}\right)+\alpha_{0}\tau_{\mathrm{int}}^{3}}{{\left(1-z^{-1}\right)}^{3}} \end{array}$$

and the closed loop transfer function as:

(11) $$\begin{array}{c} H_{c_{3}}\left(z\right)=\frac{\alpha_{2}\tau_{\mathrm{int}}{\left(1-z^{-1}\right)}^{2}+\alpha_{1}\tau_{\mathrm{int}}^{2}\left(1-z^{-1}\right)+\alpha_{0}\tau_{\mathrm{int}}^{3}}{{\left(1-z^{-1}\right)}^{3}+\alpha_{2}\tau_{\mathrm{int}}{\left(1-z^{-1}\right)}^{2}+\alpha_{1}\tau_{\mathrm{int}}^{2}\left(1-z^{-1}\right)+\alpha_{0}\tau_{\mathrm{int}}^{3}} \end{array}$$

If the integration time tends to zero ($\tau_{\mathrm{int}}\to 0$), the analog one sided equivalent loop bandwidth is equal to the digital one [29,30,31]. The relation between the analog loop bandwidth and the third-order tracking loop coefficients is well-known [1,32]:

(12) $$\begin{array}{c} B=\frac{\alpha_{2}^{2}\alpha_{1}-\alpha_{2}\alpha_{0}+\alpha_{1}^{2}}{4(\alpha_{2}\alpha_{1}-\alpha_{0})} \end{array}$$

A relation between these coefficients can simplify the previous expression [1]:

(13) $$\begin{array}{c} B_{{\mathrm{cont}}_{3\times 1}}=\left[\begin{array}{c} \alpha_{2}\\ \alpha_{1}\\ \alpha_{0} \end{array}\right]=\left[\begin{array}{c} 2.4\cdot \omega \\ 1.1\cdot \omega^{2}\\ \omega^{3} \end{array}\right] \end{array}$$

where ω is the so-called natural frequency. Further developing Equation (12), the relation between the loop bandwidth *B* and ω is achieved:

(14) $$\begin{array}{c} B=\frac{\omega }{0.7845} \end{array}$$

A change of the loop bandwidth *B* leads to an update of the natural frequency *w* that, in turn, reconfigures the loop filter coefficients (i.e., the control matrix $B_{{\mathrm{cont}}_{3\times 1}}$). The following section shows three methods that adapt the loop bandwidth of an STL sub-optimally.

## 3. Variable Loop-Bandwidth Tracking Techniques

Variable loop-bandwidth tracking techniques set a connection between the loop bandwidth and time-varying scenario conditions [7]. In dynamic scenarios, a fast loop response with a large loop bandwidth is preferred to follow the dynamics, whereas, in stationary scenarios, a noise-rejecting low loop bandwidth is appropriate. Variable loop-bandwidth tracking techniques adapt the loop bandwidth depending on the noise and signal dynamics of the tracking channel. They facilitate optimal operation for the tracking loops.

**Figure 5:**
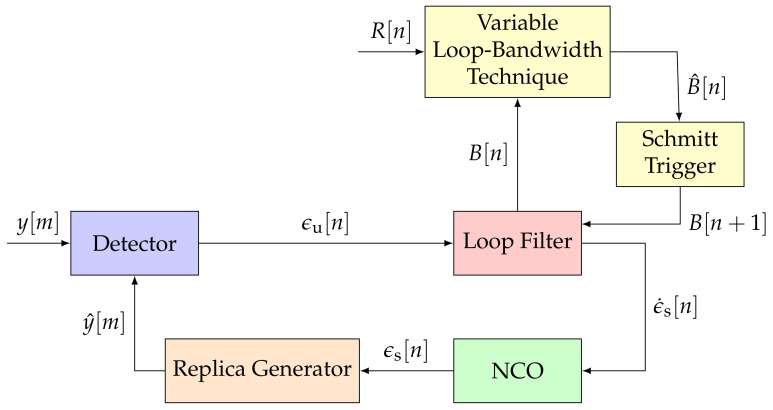
General architecture of the implemented variable loop-bandwidth tracking techniques.

Figure 5 shows the general structure of this technique [33]. The loop bandwidth B[n] and a set of measurements R[n] are the inputs to the adaptive loop-bandwidth estimator. These techniques estimate a loop bandwidth $\hat{B}\left[n\right]$. Since the set of measurements is noisy, a Schmitt trigger module reduces the noise instabilities of the loop bandwidth estimate.

The Schmitt trigger only changes the next loop bandwidth B[n+1] by ΔB if the absolute difference between the estimated loop bandwidth and the actual one exceed ΔB:

(15) $$B\left[n+1\right]=\left\{\begin{array}{cc} 0 & \mathrm{if}\;n=0\\ \hat{B}\left[n\right]+\Delta B & \mathrm{if}\;\hat{B}\left[n\right]-B\left[n\right]\geq \Delta B\;\wedge \;n>0\\ \hat{B}\left[n\right]-\Delta B & \mathrm{if}\;B\left[n\right]-\hat{B}\left[n\right]\leq \Delta B\;\wedge \;n>0\\ B[n] & \mathrm{otherwise} \end{array}\right.$$

A big ΔB value (e.g., 10 Hz) could unstable the tracking due to big loop bandwidth changes, but a very low value (e.g., 0.01 Hz) could also cause instabilities due to excessive changes. 0.5 Hz has been found an appropriate value for ΔB. Finally, the updated loop bandwidth B[n+1] changes the coefficients of the STL’s transfer function (see Equations (13) and (14)).

This section presents three techniques from this category: the FAB, the FL, and the LBCA.

### 3.1. Fast Adaptive Bandwidth (FAB)

The FAB tracking technique is a model-based approach and estimates the input signal parameters (thermal noise, phase noise, or steady state error (SSE)) of the STL [33]. A model is used to define a loop-bandwidth dependent cost function c[n,B[n]]. Setting the first derivative of the cost function to zero with respect to the loop bandwidth B[n], leads to the minimum loop bandwidth $B_{\min}$ to which the STL can handle the estimated dynamics while filtering as much noise as possible.

The three-sigma rule-of-thumb of the tracking loop error is a commonly selected cost function [6]:

(16) $$c\left[n,B\left[n\right]\right]=\sigma_{\epsilon }^{s}\left[n,B\left[n\right]\right]=\sigma_{\mathrm{thermal}}^{s}\left[n,B\left[n\right]\right]+\frac{\epsilon_{\mathrm{sse}}\left[n,B\left[n\right]\right]}{3}$$

where $\sigma_{\epsilon }^{s}$ is the jitter of the smoothed error, $\sigma_{\mathrm{thermal}}^{s}$ is the thermal noise, and $\epsilon_{\mathrm{sse}}$ is the dynamic stress error. Since $\sigma_{\epsilon }^{s}$ is loop-bandwidth dependent, the first order derivative of $\sigma_{\epsilon }^{s}$ regarding the loop bandwidth can be performed. Setting the derivative to zero, the minimum loop bandwidth $B_{\min}$ is estimated [6]:

(17) $$\begin{array}{c} \frac{\partial \sigma_{\epsilon }^{s}\left[n,B[n]\right]}{\partial B[n]}=0\to B\left[n\right]=B_{\min} \end{array}$$

The estimated minimum loop bandwidth $B_{\min}$ depends on the linear *C*/*N*_0_, the integration time $\tau_{\mathrm{int}}$, the order *p*, and the steady state line-of-sight (LOS) dynamics $\frac{\partial^{p}R}{\partial t^{p}}$.

As an example, if the FAB is implemented in a third-order PLL, the following cost function is achieved equalling to zero the first derivative of the smoothed carrier phase error jitter $\sigma_{\theta }^{s}$:

(18) $$B_{\min}=\sqrt[{7}]{\frac{{\left(2\eta^{3}\frac{\partial^{3}R}{\partial t^{3}}\right)}^{2}}{\frac{1}{C/N_{o}}\left(1+\frac{1}{2\tau_{\mathrm{int}}C/N_{o}}\right){\left(\frac{360}{2\pi }\right)}^{2}}}$$

In this case, $B_{\min}$ is dependent on the linear *C*/*N*_0_ in Hz, the integration time $\tau_{\mathrm{int}}$ in seconds and the LOS jerk dynamics $\frac{\partial^{3}R}{\partial t^{3}}$ in $\deg/s^{3}$. For a third-order PLL, the constant η equals to 0.7845. The *C*/*N*_0_ and steady state LOS dynamics must be estimated, whereas the other parameters are fixed.

Several considerations must be taken into account when implementing the FAB. First, the noisy output of the discriminator makes it difficult to measure the SSE correctly (even more for higher order loops). One solution is to accumulate the noisy measurements for a large interval [10] or to implement a dynamic error filter [11]. Second, an abrupt change of the estimated optimal loop bandwidth $B_{\min}$ may create tracking instabilities, and needs to be minimized. An empirical scaling factor for the dynamic stress estimator and a constrained loop bandwidth addresses this issue [11]. Another solution is to smooth the loop bandwidth update using the Newton-Raphson method and an IIR filter [12].

**Figure 6:**
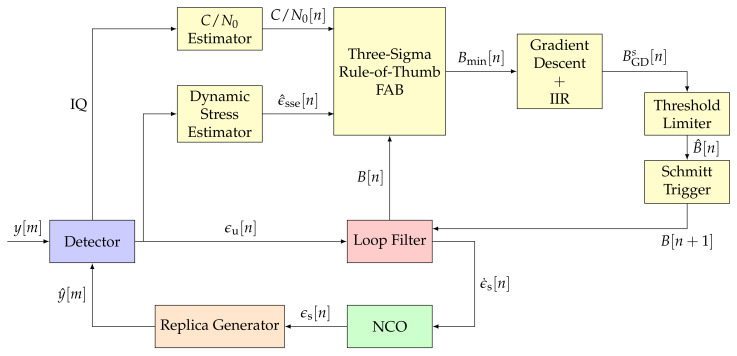
Architecture of the three-sigma rule-of-thumb based Fast Adaptive Bandwidth (FAB). ©IEEE. Adapted, with permission, from [7].

Figure 6 shows the structure of the implemented three-sigma rule-of-thumb based FAB: a *C*/*N*_0_ estimator, a dynamic stress estimator, the FAB, a gradient decent, an IIR filter and a threshold limiter. Each component is explained in the subsequent paragraphs.

The *C*/*N*_0_ estimator updates each tracking epoch. The dynamic stress estimator first filters the discriminator’s output. The filter is a first order IIR filter with a decay time of Δt. The value of Δt represents the algorithm’s sensitivity to dynamics. Second, it performs the *p*–th derivative of the filtered discriminator’s output $\mu_{\epsilon }^{u}$ in order to achieve an estimated SSE LOS dynamic measurement ${\hat{\epsilon }}_{\mathrm{sse}}$:

(19) $${\hat{\epsilon }}_{\mathrm{sse}}=\frac{\sum_{i=0}^{p}{\left(-1\right)}^{i}\left(\begin{array}{c} p\\ i \end{array}\right)\mu_{\epsilon }^{u}\left[n-i\right]}{\Delta t^{p}}$$

where $\left(\begin{array}{c} p\\ i \end{array}\right)$ is the binomial coefficient. Since the un-smoothed error is normalized, ${\hat{\epsilon }}_{\mathrm{sse}}$ is in units of (cycles/s${}^{p}$).

Continuing with the example of the third-order PLL, the dynamic stress estimator estimates the LOS jerk dynamics. This module filters the phase discriminator’s output $\mu_{\theta }^{u}$ and measures the angular jerk dynamics ${\hat{\theta }}_{\mathrm{sse}}$:

(20) $${\hat{\theta }}_{\mathrm{sse}}\left[n\right]=\frac{\mu_{\theta }^{u}\left[n\right]-3\mu_{\theta }^{u}\left[n-1\right]+3\mu_{\theta }^{u}\left[n-2\right]-\mu_{\theta }^{u}\left[n-3\right]}{\Delta t^{3}}$$

Next, the LOS jerk dynamics $\frac{\partial^{3}R}{\partial t^{3}}$ in $\deg/s^{3}$ is calculated as the following:

(21) $$\frac{\partial^{3}R}{\partial t^{3}}\left[n\right]=360^{\circ }\times {\hat{\epsilon }}_{\mathrm{sse}}\left[n\right]$$

Abrupt loop bandwidth changes may result in tracking instabilities. Therefore, the gradient descent method and a first order IIR filter smooth out the update. The gradient descent method updates the actual loop bandwidth B[n] gradually:

(22) $$B_{\mathrm{GD}}\left[n\right]=\left\{\begin{array}{cc} B\left[n\right]+\tau_{\mathrm{int}}\cdot \frac{B_{\min}\left[n\right]-B\left[n\right]}{|\Delta B_{\min}\left[n\right]|} & \mathrm{if}\;|\Delta B_{\min}\left[n\right]|>0.01\\ B_{\min}\left[n\right] & \mathrm{otherwise} \end{array}\right.$$

where $\Delta B_{\min}\left[n\right]$ is,

(23) $$\Delta B_{\min}\left[n\right]=B_{\min}\left[n-1\right]-B_{\min}\left[n\right]$$

The updated loop bandwidth $B_{\mathrm{GD}}\left[n\right]$ depends on the actual loop bandwidth B[n], the estimated minimum loop bandwidth $B_{\min}\left[n\right]$ and the previous estimated loop bandwidth $B_{\min}\left[n-1\right]$. To avoid instabilities, $B_{\mathrm{GD}}\left[n\right]$ equals $B_{\min}\left[n\right]$ when $|\Delta B_{\min}\left[n\right]|$ is below 0.01 Hz.

Next, an IIR filter smooths $B_{\mathrm{GD}}\left[n\right]$, achieving $B_{\mathrm{GD}}^{s}\left[n\right]$. Finally, the filtered loop bandwidth is passed through a threshold limiter that constraints $B_{\mathrm{GD}}^{s}\left[n\right]$ between a maximum $B_{\mathrm{Max}}$ and a minimum $B_{\mathrm{Max}}$ allowed loop bandwidth:

(24) $$\hat{B}\left[n\right]=\left\{\begin{array}{cc} B_{\mathrm{Max}} & \mathrm{if}\;B_{\mathrm{GD}}^{s}\left[n\right]\tau_{\mathrm{int}}>B_{\mathrm{Max}}\tau_{\mathrm{int}}\\ B_{\mathrm{GD}}^{s}\left[n\right] & \mathrm{if}\;B_{\mathrm{Min}}\tau_{\mathrm{int}}\leq B_{\mathrm{GD}}^{s}\left[n\right]\tau_{\mathrm{int}}\leq B_{\mathrm{Max}}\tau_{\mathrm{int}}\\ B_{\mathrm{Min}} & \mathrm{if}\;B_{\mathrm{GD}}^{s}\left[n\right]\tau_{\mathrm{int}}<B_{\mathrm{Min}}\tau_{\mathrm{int}} \end{array}\right.$$

Two main observations of the FAB can be made prior to implementation. First, the speed of the SSE estimation ${\hat{\epsilon }}_{\mathrm{sse}}$ determines the speed of the algorithm to react against signal dynamics. The standard cost function (see Equation (16)) used does not consider other sources of signal dynamics such as clock drift and low order transient dynamics. Hence, it is possible that $B_{min}$ decreases erroneously due to the fact that ${\hat{\epsilon }}_{\mathrm{sse}}$ is negligible and other dynamic sources are not included. Second, the complexity of the algorithm can be significant due to the $B_{\min}$ estimation at high-order STLs.

### 3.2. Fuzzy Logic (FL)

**Figure 7:**
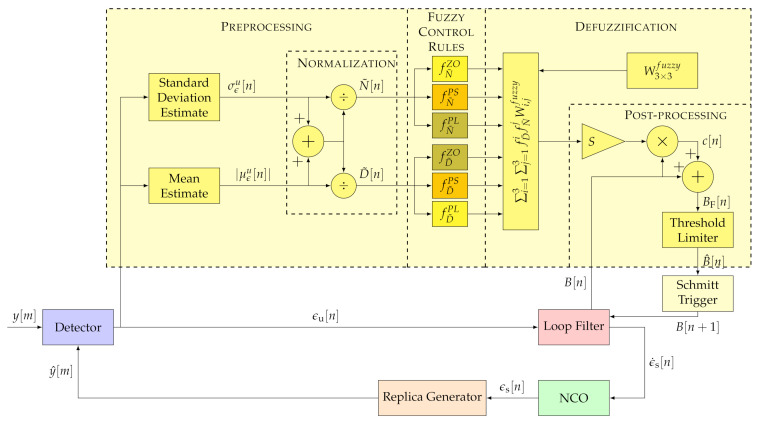
Architecture of implemented Fuzzy Logic (FL) technique. ©IEEE. Adapted, with permission, from [7].

Compared to the FAB technique, FL based tracking techniques significantly simplify the control algorithm. Figure 7 shows the implemented FL algorithm structure. The FL consists of four stages: pre-processing, fuzzy control rules, defuzzification, and post-processing [15]. The pre-processing stage converts the discriminator’s output into two fuzzy sets: The standard deviation $\sigma_{\epsilon }^{u}$ and the absolute mean $|\mu_{\epsilon }^{u}|$. The former fuzzy input represents the tracking channel’s noise, whereas the latter indicates the error dynamics. These fuzzy sets are finally normalized:

(25) $$\tilde{N}=\frac{\sigma_{\epsilon }^{u}}{\sigma_{\epsilon }^{u}+\left|\mu_{\epsilon }^{u}\right|}$$

(26) $$\tilde{D}=\frac{|\mu_{\epsilon }^{u}|}{\sigma_{\epsilon }^{u}+\left|\mu_{\epsilon }^{u}\right|}$$

where $\tilde{N}$ is the normalized estimated noise and $\tilde{D}$ the normalized estimated dynamics.

Next, the fuzzy control rules are applied. The zero $f^{\mathrm{ZO}}$, the positive-small $f^{\mathrm{PS}}$ and the positive-large $f^{\mathrm{PL}}$ linear membership functions weight each normalized estimation [15]. These fuzzy functions are characterized as follows:

(27) $$\begin{array}{cc} f^{\mathrm{ZO}}\left[n,\psi \left[n\right]\right] & =\left\{\begin{array}{cc} \frac{T_{\mathrm{Fuzzy}}^{\psi }-\psi \left[n\right]}{T_{\mathrm{Fuzzy}}^{\psi }} & \mathrm{if}\;0\leq \psi \left[n\right]\leq T_{\mathrm{Fuzzy}}^{\psi }\\ 0 & \mathrm{otherwise} \end{array}\right. \end{array}$$

(28) $$\begin{array}{cc} f^{\mathrm{PS}}\left[n,\psi \left[n\right]\right] & =\left\{\begin{array}{cc} \frac{\psi [n]}{T_{\mathrm{Fuzzy}}^{\psi }} & \mathrm{if}\;0\leq \psi \left[n\right]\leq T_{\mathrm{Fuzzy}}^{\psi }\\ \frac{1-\psi [n]}{1-T_{\mathrm{Fuzzy}}^{\psi }} & \mathrm{if}\;T_{\mathrm{Fuzzy}}^{\psi }\leq \psi \left[n\right]\leq 1\\ 0 & \mathrm{otherwise} \end{array}\right. \end{array}$$

(29) $$\begin{array}{cc} f^{\mathrm{PL}}\left[n,\psi \left[n\right]\right] & =\left\{\begin{array}{cc} \frac{\psi \left[n\right]-T_{\mathrm{Fuzzy}}^{\psi }}{1-T_{\mathrm{Fuzzy}}^{\psi }} & \mathrm{if}\;T_{\mathrm{Fuzzy}}^{\psi }\leq \psi \left[n\right]\leq 1\\ 0 & \mathrm{otherwise} \end{array}\right. \end{array}$$

where ψ[n] is the input estimation ($\tilde{N}$ or $\tilde{D}$) and $T_{\mathrm{Fuzzy}}^{\psi }$ is the function threshold that defines the regions of these three fuzzy functions. The relation between the thresholds of $\tilde{D}$ and $\tilde{N}$ is:

(30) $$T_{\mathrm{Fuzzy}}^{\tilde{N}}=1-T_{\mathrm{Fuzzy}}^{\tilde{D}}$$

**Figure 8:**
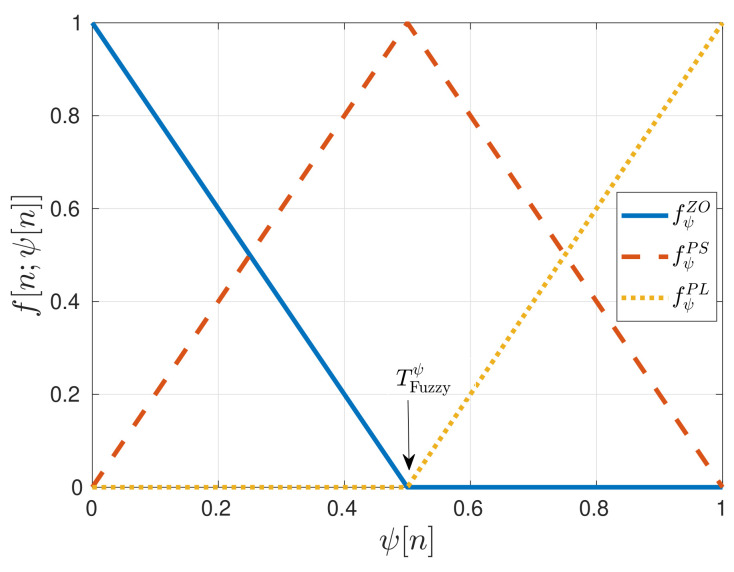
Fuzzy functions for normalized estimates $\tilde{D}$ and $\tilde{N}$. ©IEEE. Reprinted, with permission, from [7].

Figure 8 shows the fuzzy functions for $\tilde{D}$ and $\tilde{N}$. These functions have a triangular shape. The base of these triangles is so wide that it allows each fuzzy input to be a member of two sets at least. An interesting observation is the symmetry between the weighted estimates since $\tilde{D}=1-\tilde{N}$:

(31) $$f^{\mathrm{ZO}}\left[n,\tilde{D}\left[n\right]\right]=f^{\mathrm{PL}}\left[n,\tilde{N}\left[n\right]\right]$$

(32) $$f^{\mathrm{PS}}\left[n,\tilde{D}\left[n\right]\right]=f^{\mathrm{PS}}\left[n,\tilde{N}\left[n\right]\right]$$

(33) $$f^{\mathrm{PL}}\left[n,\tilde{D}\left[n\right]\right]=f^{\mathrm{ZO}}\left[n,\tilde{N}\left[n\right]\right]$$

The deffuzication stage combines the degrees of membership of each fuzzy rule. The best-known deffuzification method of the FL technique is the center of gravity (COG) [14]. The method used in this paper is similar to the fuzzy mean method (FMM) [34]. In contrast to the previous method, integration is done instead of an average. The fuzzy-weighted estimates are combined with a fuzzy weighting matrix $W_{3\times 3}^{\mathrm{fuzzy}}$:

(34) $$\begin{array}{c} P\left[n\right]=\sum_{i=1}^{3}\sum_{j=1}^{3}f^{i}\left[n,\tilde{N}\left[n\right]\right]f^{j}\left[n,\tilde{D}\left[n\right]\right]W_{i,j}^{\mathrm{fuzzy}} \end{array}$$

where $\left\{f^{1},f^{2},f^{3}\right\}$ are $\left\{f^{Z0},f^{\mathrm{PS}},f^{\mathrm{PL}}\right\}$ respectively.

The fuzzy weighting matrix $W_{3\times 3}^{\mathrm{fuzzy}}$ is a hollow matrix in which the upper triangle matrix has positive elements, and the lower triangle matrix negative values. Table 1 shows the structure of $W_{3\times 3}^{\mathrm{fuzzy}}$ [7].

The postprocessing unit scales the resultant value P[n] by *S* and multiplies it by the current loop bandwidth B[n], achieving the final control signal c[n]:

(35) $$\begin{array}{c} c[n,P[n],B[n]]=P[n]\cdot S\cdot B[n] \end{array}$$

Next, the loop bandwidth is updated:

(36) $$\begin{array}{c} B_{F}\left[n\right]=B\left[n\right]+c\left[n,P\left[n\right],B\left[n\right]\right] \end{array}$$

Finally, as the FAB technique, a threshold limiter constraints the loop bandwidth (see Equation (24)).

Two examples are addressed. If the normalized dynamics $\tilde{D}$ tends to zero, the normalized noise $\tilde{N}$ goes to one. Therefore, $f^{\mathrm{ZO}}\left[n,\tilde{D}\left[n\right]\right]$ and $f^{\mathrm{PL}}\left[n,\tilde{N}\left[n\right]\right]$ tend to one and the other fuzzy functions to zero. Consequently, P[n] is:

(37) $$P\left[n\right]=W_{3,1}^{\mathrm{fuzzy}}<0$$

The updated loop bandwidth B[n+1] decreases each iteration since the control signal is always negative. Due to the loop bandwidth decrease, the control signal c[n] increases. After some iterations, B[n] and c[n] tends to zero. This proves the lower bound stability of the algorithm.

In the opposite case, if $\tilde{D}$ tends to one, the normalized noise $\tilde{N}$ goes to zero. In such a case, $P\left[n\right]=W_{1,3}^{\mathrm{fuzzy}}>0$ and the loop bandwidth increases together with the control signal. The bigger the loop bandwidth, the bigger the update (see Equation (35)). This can lead to instabilities due to abrupt changes of the loop bandwidth. Since there is no upper bound, the same threshold limiter as in the FAB technique is implemented.

### 3.3. Loop-Bandwidth Control Algorithm

**Figure 9:**
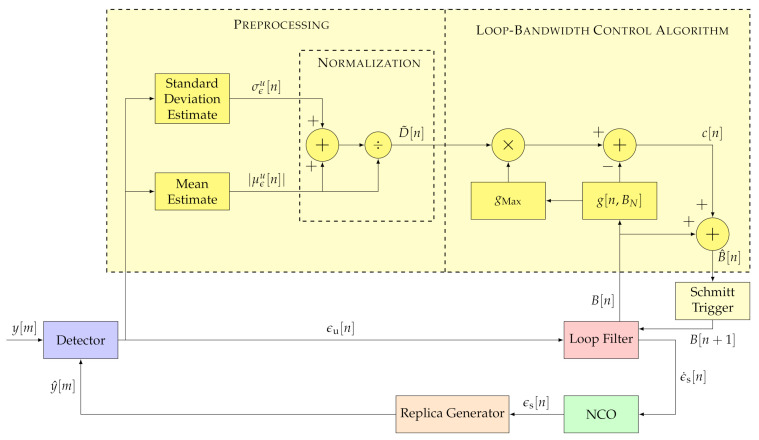
Architecture of loop-bandwidth control algorithm (LBCA). ©IEEE. Adapted, with permission, from [7].

Similar to the FL method, the LBCA [16] uses the discriminator’s statistics to adapt the loop bandwidth. However, it uses a normalized bandwidth $B_{N}$ dependent sigmoid-based weighting function to combine these values. Figure 9 shows the structure of the LBCA.

The algorithm’s inputs are the absolute mean $|\mu_{\epsilon }^{u}|$ and the standard deviation $\sigma_{\epsilon }^{u}$ estimates of the discriminator’s output. The absolute mean $|\mu_{\epsilon }^{u}|$ is interpreted as the dynamics and the standard deviation $\sigma_{\epsilon }^{u}$ as the noise of the tracking channel. The signal dynamic estimate is normalized, similar to the FL approach.

At the core, the LBCA is a weighing function $g[n,B_{N}]$. The weighting function $g[n,B_{N}]$ and the normalized dynamic estimate $\tilde{D}$, that is scaled by the maximum value of the weighting function $g_{\mathrm{Max}}$, determine the control signal $c[n,B_{N}]$:

(38) $$c\left[n,B_{N}\right]=g_{\mathrm{Max}}\cdot \tilde{D}\left[n\right]-g\left[n,B_{N}\right]$$

The control signal $c[n,B_{N}]$ and the current loop bandwidth B[n] determine the estimated loop bandwidth $\hat{B}\left[n\right]$:

(39) $$\hat{B}\left[n\right]=B\left[n\right]+c\left[n,B_{N}\right]$$

The weighting function $g[n,B_{N}]$ directly determines the adaption performance of the LBCA. It is a linear combination of *m* normalized positive sigmoid functions:

(40) $$\begin{array}{c} g\left[n,B_{N}\right]=\sum_{k=1}^{m}w_{k}{\mathrm{Sig}}_{k}\left(S_{k}\left(B_{N}\left[n\right]-P_{k}\right)\right)={\left[\begin{array}{c} w_{1}\\ \vdots \\ w_{m} \end{array}\right]}^{T}\times \left[\begin{array}{c} {\mathrm{Sig}}_{1}\left(S_{1}\left(B_{N}\left[n\right]-P_{1}\right)\right)\\ \vdots \\ {\mathrm{Sig}}_{m}\left(S_{m}\left(B_{N}\left[n\right]-P_{m}\right)\right) \end{array}\right] \end{array}$$

where $P_{k}$ is the shift parameter, $S_{k}$ is the horizontal scaling and $w_{k}$ the vertical scaling. The sigmoid function Sig(x) is defined as [35]:

(41) $$\mathrm{Sig}\left(x\right)=\frac{1}{1+e^{-x}}$$

The maximum value of the weighting function $g_{\mathrm{Max}}$ is the sum of the vertical scaling values:

(42) $$\begin{array}{c} g_{\mathrm{Max}}=\sum_{k=1}^{m}w_{k} \end{array}$$

$g_{\mathrm{Max}}$ indicates the maximum update the algorithm can perform at each iteration. It implicitly constrains the control value:

(43) $$\begin{array}{c} |c\left[n,B_{N}\right]|\leq g_{\mathrm{Max}} \end{array}$$

If the noise and signal dynamics estimates are reliable, a larger $g_{\mathrm{Max}}$ value is appropriate to facilitate faster reaction.

The shape of the weighting function $g[n,B_{N}]$ is crucial to achieving stable functionality and optimal performance. Two examples follow to illustrate the weighting function $g[n,B_{N}]$ in extreme cases. In both cases, the maximum of the weighting function is unitary ($g_{\mathrm{Max}}=1$). In the first example, a noisy static scenario causes the estimated normalized dynamics $\tilde{D}$ to tend to zero. In this case, the loop bandwidth should decrease in order to filter as much noise as possible. Therefore, the weighting function must be more significant than $\tilde{D}$ to have a negative control value. However, if the loop bandwidth approaches zero, the weighting function should also tend to zero to avoid a negative loop bandwidth and destabilize the STL. In the PLL, the loop bandwidth should be greater than a certain value to not lose lock due to clock-error frequency drifts [36]. If a temperature-compensated crystal oscillator (TCXO) is used, a good minimum normalized PLL bandwidth is around 0.06. Values less than the selected minimum normalized loop bandwidth are located in the low normalized dynamic region. In this region, the weighting function approaches zero, and the normalized bandwidth stops decreasing.

In the second example, the opposite scenario is considered. High signal dynamics at high *C*/*N*_0_, in which the normalized dynamics tend to one. Since the STL must react as fast as possible to signal dynamics, the loop bandwidth should increase. Consequently, the weighting function is lower than $\tilde{D}$ in order to have a positive control value c[n]. A condition is that the analog to digital mapping does not hold anymore if the normalized bandwidth is bigger than 0.4 [29,30,31]. Therefore, values bigger than 0.4 are located in the high normalized dynamic region.In this region the weighting function is close to one, meaning that the loop bandwidth can no longer increase.

The mentioned extreme scenarios are helpful to achieve a first glance of the weighting function’s shape. However, intermediate scenarios in which the signal dynamics and noise are equivalent must also be noted. These cases occur in the transient region.The value of the weighting function in this region is the normalized dynamic threshold $T_{\mathrm{LBCA}}$. $T_{\mathrm{LBCA}}$ determines the sensitivity to normalized dynamics. The higher the threshold, the less sensitive to signal dynamics.

**Figure 10:**
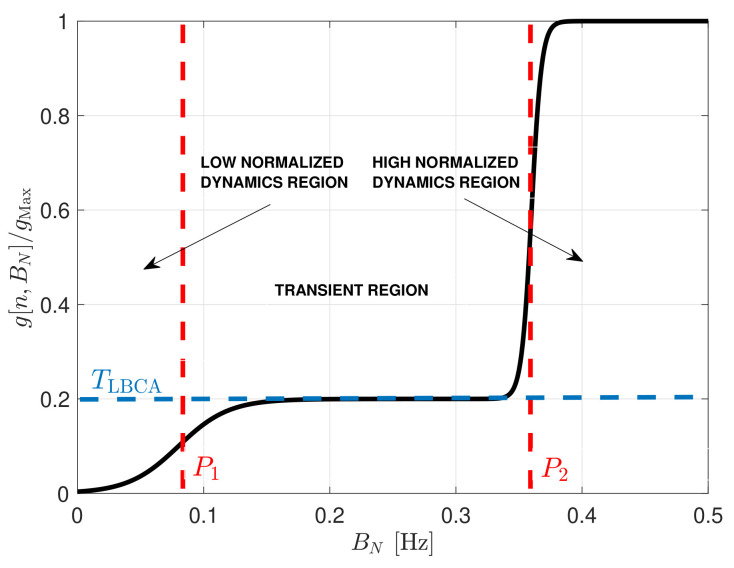
Weighting function of LBCA.

Figure 10 shows the shape of the weighting function being a linear combination of two sigmoid functions.

(44) $$g\left[n,B_{N}\right]={\left[\begin{array}{c} w_{1}\\ w_{2} \end{array}\right]}^{T}\times \left[\begin{array}{c} \mathrm{Sig}\left(S_{1}\left(B_{N}-P_{1}\right)\right)\\ \mathrm{Sig}\left(S_{2}\left(B_{N}-P_{2}\right)\right) \end{array}\right]$$

The biases $P_{1},P_{2}$ determine the borders of the regions, the horizontal scaling $S_{1},S_{2}$ indicate the slope of the transition between regions, and the vertical scaling $w_{1},w_{2}$ define the sensitivity to normalized dynamics.

The weighted function’s complexity is significant due to the sigmoid function in which a division of an exponential is required. An approach to reduce the complexity is to perform an approximation using the piecewise linear approximation of nonlinearities (PLAN) technique [37]:

(45) $${\mathrm{Sig}}_{PLAN}\left(S_{k}\left(B_{N}-P_{k}\right)\right)=\left\{\begin{array}{cc} 1 & \mathrm{if}\;S_{k}\left(B_{N}-P_{k}\right)\geq 5\\ 0.03125\cdot S_{k}\left(B_{N}-P_{k}\right)+0.84375 & \mathrm{if}\;2.375\leq S_{k}\left(B_{N}-P_{k}\right)<5\\ 0.125\cdot S_{k}\left(B_{N}-P_{k}\right)+0.625 & \mathrm{if}\;1\leq S_{k}\left(B_{N}-P_{k}\right)<2.375\\ 0.25\cdot S_{k}\left(B_{N}-P_{k}\right)+0.5 & \mathrm{if}\;0\leq S_{k}\left(B_{N}-P_{k}\right)<1\\ 1-{\mathrm{Sig}}_{PLAN}\left(S_{k}\left(B_{N}-P_{k}\right)\right) & \mathrm{if}\;S_{k}\left(B_{N}-P_{k}\right)<0 \end{array}\right.$$

**Figure 11:**
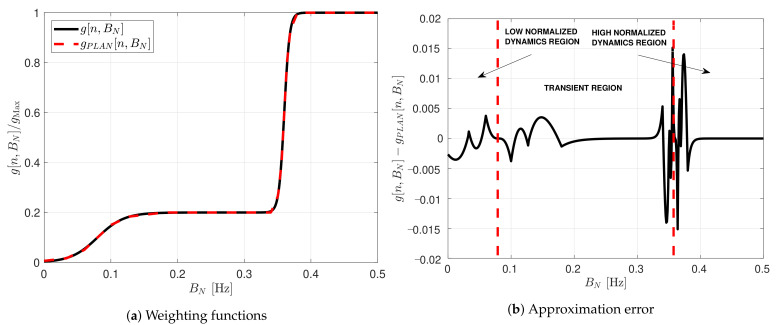
Comparison between sigmoid weighting function and its piecewise linear approximation.

Figure 11 shows the comparison between the weighting function and its piece-wise linear approximation. The approximation errors are located in the limits between regions. However, it is a good approximation since there is maximum error of 0.15 %. In the next section, both weighted functions are evaluated.

## 4. Evaluation Method of Adaptive Loop-Bandwidth Tracking Techniques

This section analyses the most adequate quality factors that represent the performance and the complexity of adaptive tracking techniques.

### 4.1. Tracking Performance

Two parameters that define an adaptive tracking technique’s tracking performance are considered: the Cramér-Rao bound (CRB) and the one-sigma rule threshold of the unsmoothed error $\sigma_{\epsilon_{u}}^{th}$. The CRB indicates the minimum error variance of an unbiased estimator [38], whereas the one-sigma rule threshold is a conservative threshold that ensures a stable tracking lock if the tracking error is less than this threshold [1]. The standard deviation of the discriminator’s error $\sigma_{\epsilon }^{u}$ is a good performance metric [8]. The tracking performance is defined as the difference between the average $\sigma_{\epsilon }^{u}$ and the lower bound standard deviation $\sigma_{\mathrm{LB}}^{u}$:

(46) $$P_{\mathrm{Tracking}}={\bar{\sigma }}_{\epsilon }^{u}-\sigma_{\mathrm{LB}}^{u}$$

The average of $\sigma_{\epsilon }^{u}$, ${\bar{\sigma }}_{\epsilon }^{u}$, is defined as:

(47) $${\bar{\sigma }}_{\epsilon }^{u}=\sum_{n=0}^{K_{\mathrm{sim}}}\frac{\sigma_{\epsilon }^{u}\left[n\right]}{K_{\mathrm{sim}}}$$

where $K_{\mathrm{sim}}$ is the discrete simulation time in samples. $K_{\mathrm{sim}}$ is represented as the product between the simulation time $T_{\mathrm{sim}}$ in seconds and the logging data rate $f_{s}$ in Hz:

(48) $$K_{\mathrm{sim}}=T_{\mathrm{sim}}f_{s}$$

**Figure 12:**
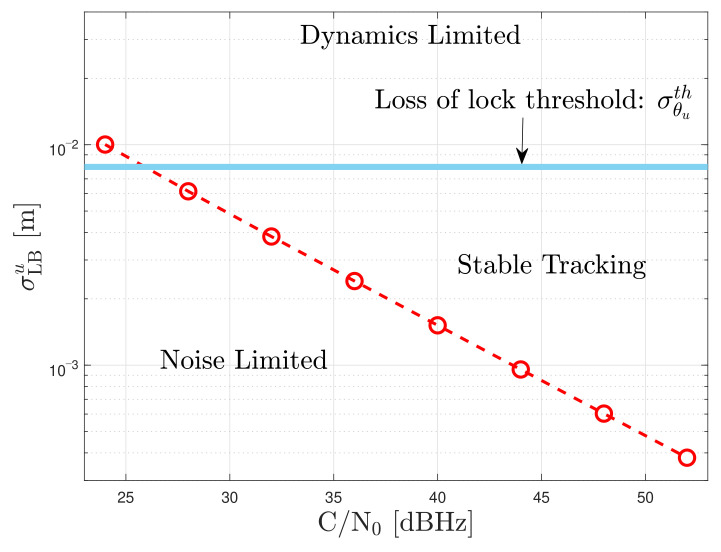
Square root CRB of the un-smoothed carrier phase error at different *C*/*N*_0_ levels, $\tau_{\mathrm{int}}=20\;\mathrm{ms}$.

Since the adaptive tracking techniques are implemented in a third-order Costas PLL, the PLL tracking performance is analyzed. Considering the PLL as a time of arrival (ToA) unbiased estimator [39], the resulting square-root CRB of the un-smoothed carrier phase error $\theta_{u}$ in meters is:

(49) $$\sigma_{\mathrm{LB}}^{u}=\left(\frac{\lambda }{2\pi }\right)\sqrt{\frac{1}{2\tau_{\mathrm{int}}C/N_{0}}\left(1+\frac{1}{2\tau_{\mathrm{int}}C/N_{0}}\right)}$$

where λ is the wavelength of the GNSS signal and the term $1+\frac{1}{2\tau_{\mathrm{int}}C/N_{0}}$ is the squaring loss. Figure 12 shows the lower bound of the un-smoothed carrier phase error with an integration time $\tau_{\mathrm{int}}$ of 20 ms at different *C*/*N*_0_ levels.

From the three-sigma rule-of-thumb, the carrier phase error jitter $\sigma_{\theta }^{s}$ must be less than a conservative threshold to ensure stable tracking and no cycle-slips [1]. This upper threshold is also applied to the un-smoothed phase error jitter $\sigma_{\theta }^{u}$. Since a two-quadrant arctangent discriminator is used, the one-sigma rule threshold in meters is:

(50) $$\sigma_{\theta^{u}}^{th}=\frac{1}{3}\times \frac{\Omega }{4}\times \frac{\lambda }{2\pi }=\frac{\pi }{12}\times \frac{\lambda }{2\pi }=\frac{\lambda }{24}$$

where Ω is the phase pull-in range in radians. The 1/12 factor in Eqution (50) is included because the one-sigma rule threshold is one-third of the three-sigma rule threshold and one-fourth of Ω is selected to have a conservative threshold.

### 4.2. System Performance

The system performance considers the performance of all the tracking channels together. The average lock indicator for each tracking channel is an appropriate metric to measure the system performance in terms of precision [40,41].

Since this study evaluates the performance of the PLL, the phase-lock indicator (PLI) is considered:

(51) $$\mathrm{PLI}=\frac{I_{p}^{2}-Q_{p}^{2}}{I_{p}^{2}+Q_{p}^{2}}$$

where $I_{p}$ and $Q_{p}$ are the in-phase and quadrature prompt correlation values.

The selected quality factor is the average of the PLI with respect the discrete simulation time $K_{\mathrm{sim}}$ and the accumulated number of tracked satellite vehicles (SVs) $N_{\mathrm{sat}}^{\mathrm{Acc}}$:

(52) $$\bar{\mathrm{PLI}}=\sum_{l=0}^{N_{\mathrm{sat}}^{\mathrm{Acc}}}\sum_{n=0}^{K_{\mathrm{sim}}}\frac{{\mathrm{PLI}}^{l}\left[n\right]}{K_{\mathrm{sim}}N_{\mathrm{sat}}^{\mathrm{Acc}}}$$

If a SV is tracked at least once during the simulation, the PLI of this SV is considered to calculate $\bar{\mathrm{PLI}}$.

In order to measure the system performance in terms of robustness against dynamics, a good quality factor is the average number of visible satellites being tracked ${\bar{N}}_{\mathrm{sat}}$:

(53) $$\begin{array}{c} {\bar{N}}_{\mathrm{sat}}=\sum_{n=0}^{K_{\mathrm{sim}}}\frac{N_{\mathrm{sat}}\left[n\right]}{K_{\mathrm{sim}}N_{\mathrm{sat}}^{\mathrm{Total}}} \end{array}$$

where $N_{\mathrm{sat}}^{\mathrm{Total}}$ is the total number of visible satellites in the scenario.

A final metric is achieved combining (52) and (53):

(54) $$\begin{array}{c} P_{\mathrm{System}}=\bar{\mathrm{PLI}}\times {\bar{N}}_{\mathrm{sat}} \end{array}$$

The average system performance ${\bar{P}}_{\mathrm{System}}$ with respect the *C*/*N*_0_ levels determines the overall performance of the adaptive tracking technique.

(55) $$\begin{array}{c} {\bar{P}}_{\mathrm{System}}=\sum_{k=1}^{N_{\mathrm{CN}0}}\frac{P_{\mathrm{System}}^{k}}{N_{\mathrm{CN}0}} \end{array}$$

where $N_{\mathrm{CN}0}$ is the number of *C*/*N*_0_ levels. The system performance metric ${\bar{P}}_{\mathrm{System}}$ which accounts for both noise and dynamics for tracking, is a novel contribution of this paper.

### 4.3. Complexity

A theoretical method to quantify an adaptive tracking technique’s complexity is to measure the number of required mathematical operations. This method provides a “best-case” comparison, and neglects any implementation limitations. Table 2 shows the theoretical number of additions, multiplications, divisions and different operations required for each case. The total number of operations for each adaptive tracking technique is labeled in colors from the highest, in red, to the lowest number of operations, in green.

Since the algorithms are implemented in software, the time complexity is a good practical complexity indicator. The time complexity measures the processing time the algorithm takes in software on the processing platform. Although this approach depends on how exemplary the algorithm’s software implementation is, the results show a correlation between the number of operations and the time complexity. The time complexity is measured in an Intel Skylake micro-architecture with a clock speed of 3700 MHz. The code is implemented in C++, and a for-loop is used to iterate the operation of a loop filter $3\times 10^{8}$ times. The *chrono* library is used to measure the processing time of the algorithm [42]. Also, the performance profiler tool for Linux *operf* serves to have a statistical report of the libraries used during the algorithm’s execution [43]. In order to avoid multi-threading, the *taskset* command is used to bind the application process to one single core [44]. Table 3 shows the total time complexity $T_{C}$ in seconds, the average time complexity at each iteration $T_{\mathrm{Iter}}$ in nanoseconds, and the added time complexity $T_{\mathrm{Added}}$ compared to the standard loop filter $T_{C}^{\mathrm{Standard}}$ (i.e., with no adaption algorithms). The $T_{\mathrm{Added}}$ values are labeled in colors from the most complex algorithm, in red, to the less complex one, in green. The iteration time complexity is defined as:

(56) $$\begin{array}{c} T_{\mathrm{Iter}}=\frac{T_{C}}{3\times 10^{8}}\times 10^{9}\;\left(\mathrm{nanoseconds}\right) \end{array}$$

and the added time complexity is expressed as:

(57) $$\begin{array}{c} T_{\mathrm{Added}}=\frac{T_{C}}{T_{C}^{\mathrm{Standard}}}\left(\mathrm{times}\right) \end{array}$$

The FAB technique presents high complexity due to the seventh root (see Equation (18)). This is verified from the profiler report, which shows high use of the *pow* function from the *libm* library [45]. The derivation of the Newton-Raphson method [46] can approximate this operation in order to achieve a lower complexity. However, this approximation is not performed in the actual implementation. The FL technique presents a lower time complexity than the FAB technique but higher than the LBCA. The profiler report of the LBCA shows the use of the *exp* function from the *libm* library [45]. The piece-wise linear approximation of the weighting function in the LBCA technique removes that function’s utilization, reducing the algorithm’s complexity.

## 5. Experimental Setup

This section describes the experimental setup and the expected results.

### 5.1. Receiver and Algorithm Implementation

**Figure 13:**
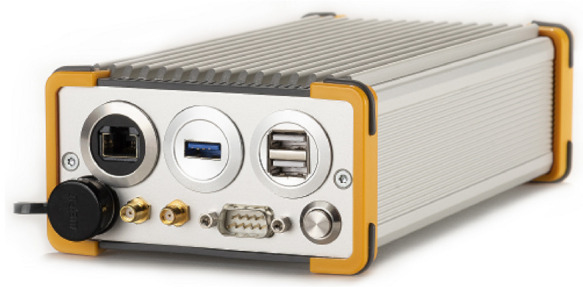
Photo of the GOOSE receiver @Fraunhofer IIS/Paul Pulkert.

The GOOSE© platform, developed by Fraunhofer IIS and marketed through TeleOrbit GmbH, is a GNSS receiver with an open software interface [24,25]. A picture of the receiver is shown in Figure 13. The receiver is based on a Xilinx Kintex7 field-programmable gate array (FPGA), depicted in green, connected to an external processor, in purple, using a peripheral component interconnect express (PCIe) interface. The FPGA receives digital samples from a customized tri-band radio-frequency front-end (RFFE), in red. One acquisition module and sixty tracking channels are implemented, which can be controlled by an external processor.

**Figure 14:**
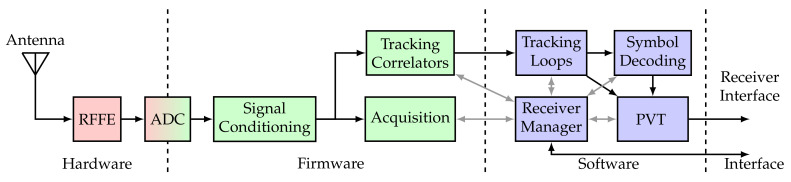
GOOSE architecture diagram.

Processing intense portions of the tracking stage are implemented in hardware (“Tracking Correlators”) and the remainder in software (“Tracking Loops”). The correlators and the NCOs are implemented on an FPGA, whereas the software part includes the discriminators, the loop filters, and the adaptive tracking algorithms. Figure 14 shows a block diagram of the entire receiver. The analog hardware is shown in red, the digital firmware in green, and the digital software in blue. It also contains the RFFE, an analog-to-digital converter (ADC), signal conditioning, acquisition, the receiver manager, symbol decoding, and the PVT engine.

Each tracking channel contains a second-order FLL, a third-order Costas PLL and a second-order DLL with PLL-assisted-DLL (PAD) enabled. The FLL is first enabled in order to track and refine the acquired Doppler frequency. Once a stable frequency lock is achieved, the transition to the FLL-assisted-PLL (FAP) is done. In this stage the PLL lock indicator is stabilizing with the assistance of the FLL. After obtaining a good and stable PLL lock, the FLL gets disabled in order to improve the tracking accuracy. In this final stage, the bit synchronization is performed to decode the navigation message and get a PVT solution. If the PLL lock deteriorates, the receiver goes back to the previous stage FAP by default in order to recover the carrier lock. However, for these tests this condition is disabled in order to evaluate correctly the adaptive carrier phase tracking capabilities.

The purpose is to evaluate the performance and the complexity of each variable loop-bandwidth tracking technique implemented in this receiver’s PLL against simulated scenarios with different dynamics and noise levels. In this study, only Global Positioning System (GPS) L1 C/A signals are considered. Moreover, the algorithms are evaluated at an integration time $\tau_{\mathrm{int}}$ of 20 ms. Table 4 shows the initial configuration of the DLL, FLL, and PLL that is used for all the tests.

The FAB, the FL, the LBCA, and the LBCA with PLAN algorithms are implemented on the GOOSE receiver in software. These adaptive tracking techniques are configured to adapt the loop bandwidth of a third-order Costas PLL.

#### 5.1.1. FAB Configuration

Since the integration time $\tau_{\mathrm{int}}$ is 20 ms, a loop bandwidth bigger than 18 Hz could destabilize the tracking because of the wrong mapping from analog to digital domain [29,30,31]. Hence, the selected upper bound $B_{\mathrm{Max}}$ of the threshold limiter (see Equation (24)) is set to 18 Hz. It has been observed that a PLL loop bandwidth lower than 4 Hz cannot remain in lock, probably because of permanent signal dynamics due to nonlinearities. Consequently, in case the estimation tends erroneously to zero, a lower bound 4 Hz is set in the threshold limiter.

(58) $$\hat{B}\left[n\right]=\left\{\begin{array}{cc} 18 & \mathrm{if}\;B_{\mathrm{GD}}^{s}\left[n\right]>18\\ B_{\mathrm{GD}}^{s}\left[n\right] & \mathrm{if}\;4\leq B_{\mathrm{GD}}^{s}\left[n\right]\leq 18\\ 4 & \mathrm{if}\;B_{\mathrm{GD}}^{s}\left[n\right]<4 \end{array}\right.$$

In the following section, the FAB algorithm’s performance is evaluated in terms of the filtered time Δt of the steady state error dynamics estimator (see Equation (20)). This parameter is selected since it directly affects the algorithm’s sensitivity to dynamics.

#### 5.1.2. FL Configuration

Table 5 shows the empirically selected values of the fuzzy matrix $W_{3\times 3}^{\mathrm{fuzzy}}$ [7].

The same threshold limiter as in the FAB technique is used (see Equation (58)). Another parameter to consider is the fuzzy threshold $T_{\mathrm{Fuzzy}}^{\psi }$. Based on the three-sigma rule-of-thumb, the optimal normalized dynamics can be calculated [16]. $D^{\mathrm{opt}}$ is the optimal normalized dynamics in order to achieve best tracking performance. For a third-order PLL the optimal normalized dynamics $D^{\mathrm{opt}}$ is 1/7. In the following section, the FL technique’s performance is evaluated, varying the normalized dynamics fuzzy threshold $T_{\mathrm{fuzzy}}^{\tilde{D}}$ around $D^{\mathrm{opt}}$. Moreover, the effects of the scale factor *S* is shown.

#### 5.1.3. LBCA Configuration

The selected weighting function is a linear combination of two sigmoid functions and has the following expression:

(59) $$g\left[n,B_{N}\right]=S{\left[\begin{array}{c} T_{\mathrm{LBCA}}\\ 1-T_{\mathrm{LBCA}} \end{array}\right]}^{T}\times \left[\begin{array}{c} \mathrm{Sig}\left(50(B_{N}-0.06)\right)\\ \mathrm{Sig}\left(250(B_{N}-0.36)\right) \end{array}\right]$$

The biases constraints the normalized bandwidth $B_{N}$. The algorithm cannot go below a $B_{N}$ lower than 0.06. In theory, the lower limit should be set at zero if there is no signal dynamics. However, it is considered that there is always some dynamics due to the clock drift or other non-linearities. Therefore, 0.06 is chosen as a lower limit. The upper limit is chosen due to the mapping limitation from the analog domain to the digital domain. The empirically selected horizontal scaling values have an adequate slope in the borders between regions. The vertical scaling depends on the normalized dynamics threshold $T_{\mathrm{LBCA}}$, and a scale factor *S*. The former parameter determines the sensitivity to dynamics, whereas the latter indicates the maximum loop bandwidth update of the algorithm. *S* is equivalent to $g_{\mathrm{Max}}$:

(60) $$g_{\mathrm{Max}}=S\cdot \left(T_{\mathrm{LBCA}}+1-T_{\mathrm{LBCA}}\right)=S$$

As in the FL configuration, $T_{\mathrm{LBCA}}$ must be around the optimal normalized dynamics $D^{\mathrm{opt}}$. Different normalized dynamics threshold $T_{\mathrm{LBCA}}$ values and scale factors $g_{\mathrm{Max}}$ are presented in the following section.

### 5.2. Evaluation Setup

**Figure 15:**
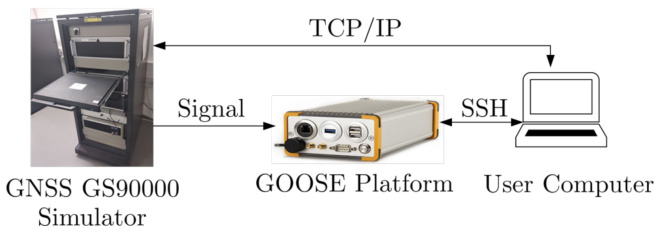
Setup for the simulation. ©IEEE. Adapted, with permission, from [7].

Figure 15 presents the test set-up. It is the same as with previous studies [16], where a Spirent GSS9000 radio-frequency constellation simulator (RFCS) generates controlled scenarios. The simulation duration is 20 min and the simulation repeats for different *C*/*N*_0_ levels. In this case, the simulation repeats 8 times, from 24 dBHz to 52 dBHz in 4 dB steps. Since the sensitivity of the acquisition is lower than the sensitivity of the tracking, the simulation always starts at the highest *C*/*N*_0_ level, 52 dBHz. The *C*/*N*_0_ level is reduced by 4 dB in 30 s intervals, until reaching the desired level. For example, 3.5 min are necessary to reach a *C*/*N*_0_ level of 24 dBHz. Therefore, in order to assure that the measured tracking and system performance is reliable, the last 10 min of the simulation are considered, $T_{\mathrm{sim}}=600$ s. Only GPS L1 C/A signals are generated by the RFCS, as only these signals are evaluated in this study. However, for future evaluations, other GNSS signals will also be considered.

**Figure 16:**
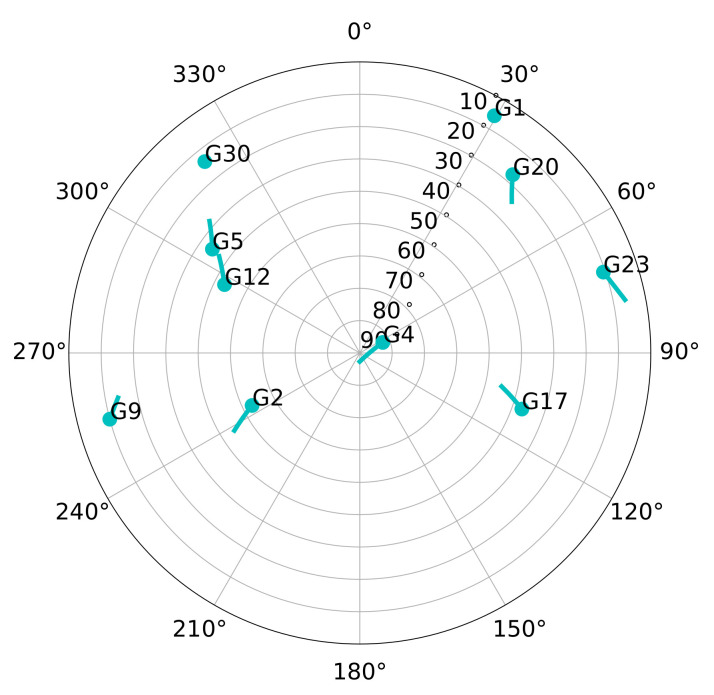
Sky-plot of simulated scenario.

The generated scenarios are either static or have receiver dynamics. The static scenarios represent stationary use-cases such as GNSS reference stations, whereas the dynamic scenarios represent harsh vehicular conditions. Figure 16 shows the sky-plot of both scenarios. There are 10 visible satellites during the simulation. However, SV G1 disappears behind the horizon after two minutes of simulation and SV G30 rises above the horizon near the end of the simulation. Therefore, the maximum number of visible satellites $N_{\mathrm{sat}}^{\mathrm{Total}}$ is limited to eight.

**Figure 17:**
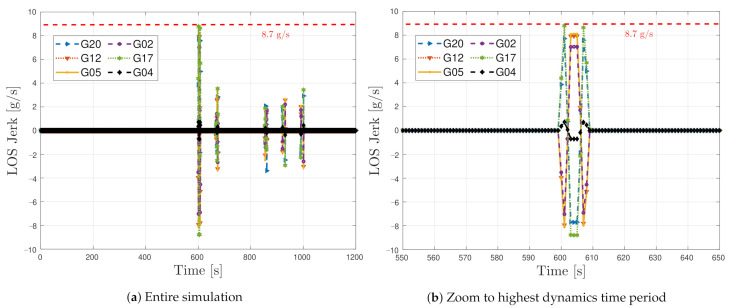
Jerk line-of-sight dynamics of simulated dynamic scenario.

Figure 17 shows the LOS jerk dynamics of six out of the eight GPS SVs in the simulated dynamic scenario. The first 10 min of the simulated dynamic scenario is static. In this way, the adaptive tracking converges to a steady estimation of the loop bandwidth equal to the one in the simulated static scenario. After 10 min of simulation, the high dynamic vehicular scenario begins. The simulated trajectory presents the highest LOS dynamics at low elevation satellites, whereas high elevation ones have reduced dynamics. For example, SV G4 is located in the zenith, and a maximum LOS jerk of 1 g/s is observed, whereas SV G17 presents the highest LOS jerks dynamics with 8.7 g/s. Figure 17b focuses on the time with the highest dynamics. In this period, the tracking of most of the satellites will be lost.

Before evaluating the adaptive tracking techniques, the tracking and system performance of the traditional PLL are measured. This helps to observe the performance improvement of adaptive loop-bandwidth tracking techniques compared to standard tracking. A single tracking channel is tested to evaluate the tracking performance, and the tracking outputs, including the correlator IQ values, discriminator outputs, current loop bandwidth, and the estimated *C*/*N*_0_, are logged. For the system performance, all the eight visible GPS SVs are considered and the open GNSS receiver protocol (OGRP) is used for logging the receiver measurements [47,48]. The average system performance ${\bar{P}}_{\mathrm{System}}$ metric for the standard PLL and the adaptive algorithms are calculated (see Equation (55)). Lastly, basic Pareto optimization [49,50,51] is applied to identify the best techniques based on the static ${\bar{P}}_{\mathrm{System}}$ and dynamic ${\bar{P}}_{\mathrm{System}}$ trade-off.

The data rate $f_{s}$ of the logged measurements equals $1/\tau_{\mathrm{int}}$. Since the integration time $\tau_{\mathrm{int}}$ is 20 ms, the data rate is 50 Hz. Therefore, considering Equation (48) and $T_{\mathrm{sim}}=600$ s, the discrete simulation time $K_{\mathrm{sim}}$ equals 30,000 samples.

## 6. Results

The results are separated into four sections. First, the static performance is evaluated to show tracking capabilities in noise only environments. Second, dynamic performance is measured to show the capabilities for vehicular applications. Third, the synthesis of all the gathered results is performed. The last section presents a final comparison between system performance and time complexity. The dataset used to plot the presented results are available on the cloud [52].

### 6.1. Static Scenario

**Figure 18:**
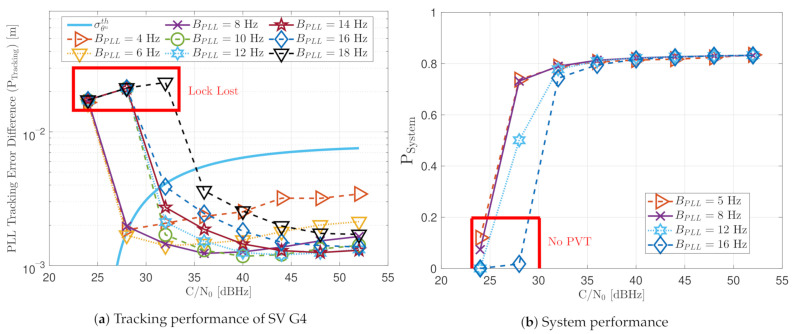
Static performance of standard PLL at different *C*/*N*_0_ levels and loop bandwidths.

Figure 18 shows the GNSS-Receiver with Open Software Interface (GOOSE) receiver’s tracking and system performance in a static scenario at different PLL loop bandwidths and *C*/*N*_0_ levels. Figure Figure 18a presents the tracking performance of SV G4. The one-sigma rule threshold $\sigma_{\theta^{u}}^{th}$ in meters is also included. Values greater than $\sigma_{\theta^{u}}^{th}$ indicate that the lock of the tracking is likely lost and experiencing severe cycle slips. Therefore, only values below this threshold can be regarded as being on a stable track. The tracking performance at different loop bandwidths differs in order of millimeters. The results have the same tendency as previous results obtained by another GOOSE receiver [16]. At a higher *C*/*N*_0_ level, a bigger loop bandwidth reduces the tracking error difference. This is due to the fast response of the filter to correct the carrier phase error. On the contrary, if the *C*/*N*_0_ decreases, a lower loop bandwidth is adequate to reduce the error’s noise.

The system performance $P_{\mathrm{System}}$ is the product between the average PLI, $\bar{\mathrm{PLI}}$, and the average number of tracked visible satellites ${\bar{N}}_{\mathrm{sat}}$ (see Equation (54)). Figure 18b shows the system performance of the GOOSE receiver at different *C*/*N*_0_ levels. At high *C*/*N*_0_ values, all bandwidths achieve good performance. However, at low *C*/*N*_0_, broader loop bandwidths result in poor performance. This shows how effective a narrow loop bandwidth can suppress noise effects.

**Figure 19:**
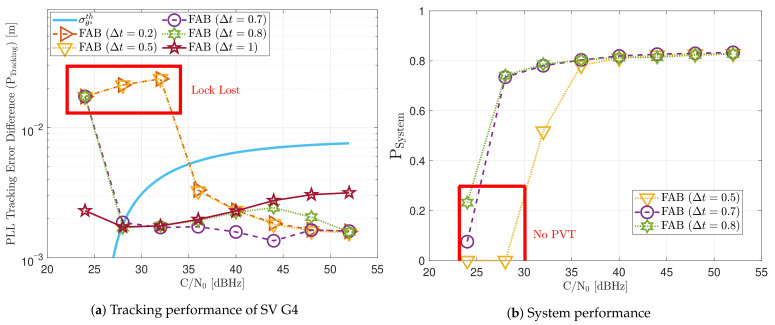
Static performance of FAB PLL at different *C*/*N*_0_ levels.

Figure 19 includes the tracking performance of SV G4 using the FAB PLL (see Figure 19a) and the system performance considering all the visible SVs (see Figure 19b). The FAB technique changes its sensitivity to dynamics depending on the IIR filter decay time Δt to estimate the SSE (see Equation (20)). Hence, different decay times are evaluated to see how this parameter affects the static performance. If the time of filtered data is less than 0.5 s, the algorithm is too sensitive to dynamics, and the tracking performance deteriorates at low *C*/*N*_0_. From a system perspective, this configuration is the only one that does not achieve PVT at 28 dBHz. The system performance is equivalent to the other configurations at higher *C*/*N*_0_ levels. If the decay time Δt is above 0.8 s, the tracking performance deteriorates at higher *C*/*N*_0_ level. An intermediate Δt such as 0.7 s seems to have the best tracking performance. Therefore, an adequate IIR filter with a decay time Δt is determined to be between 0.6 s and 0.8 s.

**Figure 20:**
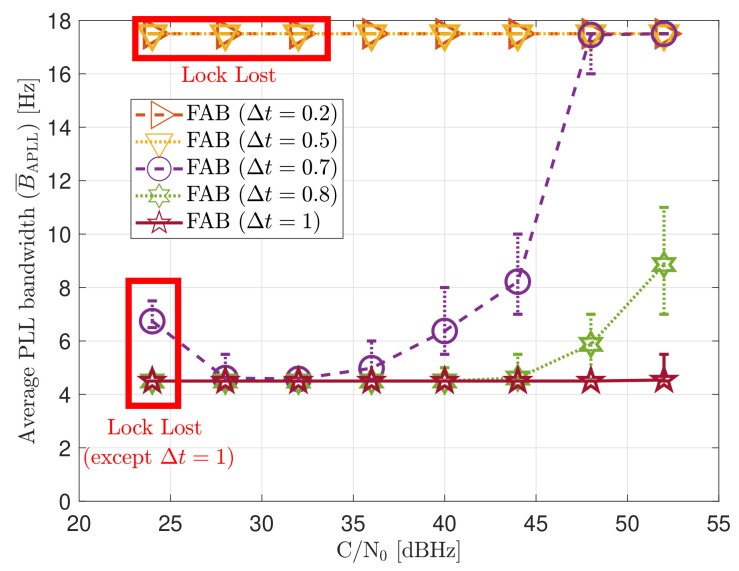
Average FAB PLL loop bandwidth of SV G4 trackingin static scenario.

Figure 20 shows the average loop bandwidth of the adaptive FAB PLL ${\bar{B}}_{\mathrm{APLL}}$. The average loop bandwidth is calculated as:

(61) $${\bar{B}}_{\mathrm{APLL}}=\sum_{k=0}^{K_{\mathrm{sim}}}\frac{B_{{\mathrm{APLL}}_{k}}}{K_{\mathrm{sim}}}$$

The added error bars indicate the maximum and minimum estimated loop bandwidth during the entire simulation. If Δt is less than 0.5 s, the average loop bandwidth is around 17.5 Hz for all the *C*/*N*_0_ levels. The estimated loop bandwidth goes beyond 18 Hz, but the threshold limiter constraints it to 17.5 Hz in order to avoid destabilizing the tracking. On the contrary, if the decay time Δt is above 0.8 s, the algorithm does not detect any steady-state dynamics. Therefore, the estimated loop bandwidth tends to zero. However, the threshold limiter constraints the loop bandwidth to 4.5 Hz.

**Figure 21:**
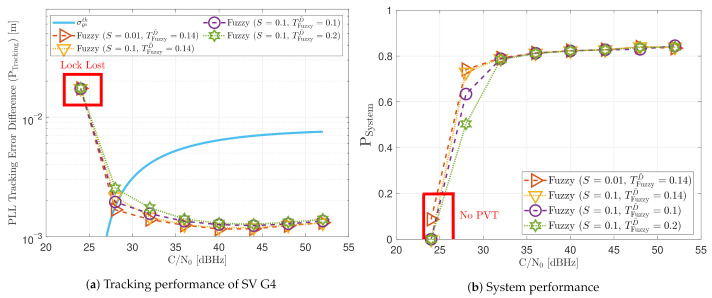
Static performance of FL PLL at different *C*/*N*_0_ levels.

Figure 21 shows the static performance of the FL PLL. The normalized dynamic function threshold $T_{Fuzzy}^{\tilde{D}}$ defines the sensitivity to dynamics, and the scale factor *S* indicates the speed of the update rate. Different function thresholds and scale factors are selected to evaluate the FL. At high *C*/*N*_0_ levels, each configuration’s tracking error performance is similar (see Figure 21a). Conversely, at lower *C*/*N*_0_, the tracking performance degrades if the FL is too sensitive ($T_{Fuzzy}^{\tilde{D}}=0.1$) or too insensitive ($T_{Fuzzy}^{\tilde{D}}=0.2$) to signal dynamics. An intermediate threshold such as $T_{Fuzzy}^{\tilde{D}}=0.14$ results in better tracking performance. The same behaviour is observed in the system performance (see Figure 21b). At 28 dBHz, $T_{Fuzzy}^{\tilde{D}}=0.1$ and $T_{Fuzzy}^{\tilde{D}}=0.2$ presents worse performance than $T_{Fuzzy}^{\tilde{D}}=0.14$.

**Figure 22:**
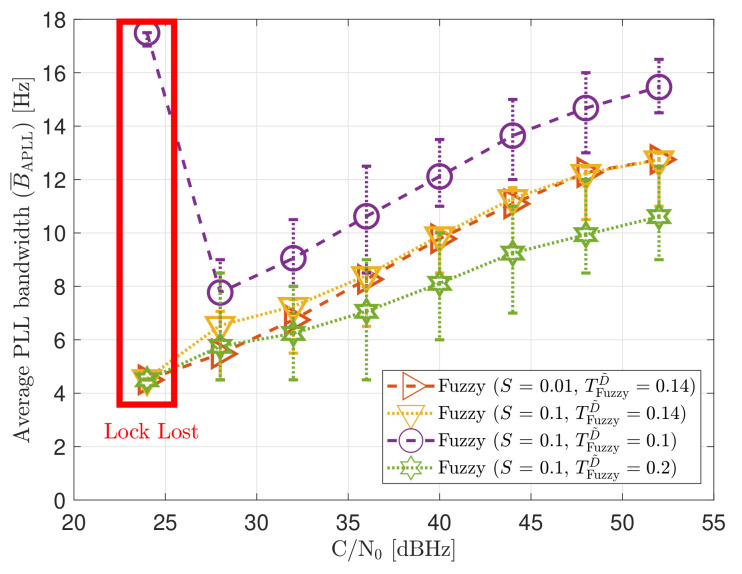
Average FL PLL loop bandwidth of SV G4 tracking in static scenario.

Figure 22 reveals that a decrease in $T_{Fuzzy}^{\tilde{D}}$ leads to a bigger average loop bandwidth ${\bar{B}}_{\mathrm{APLL}}$. If the scale factor *S* increases, the maximum and minimum value of the estimated loop bandwidth also rise. This indicates that the loop bandwidth oscillates over a broader range, and it can be a problem at low *C*/*N*_0_ levels. Another interesting observation is the algorithm’s behavior once the lock is lost. For instance, at 24 dBHz, all the presented configurations lose the tracking lock. On the one hand, if the configuration is too sensitive to signal dynamics (e.g., $T_{Fuzzy}^{\tilde{D}}=0.1$), the loss of lock is interpreted as signal dynamics, and the loop bandwidth increases at each iteration. Since there is a threshold limiter, the loop bandwidth is constrained to the upper limit, 17.5 Hz. On the other hand, if the configuration is less sensitive to dynamics, the loop bandwidth correctly decreases to zero when there is a lock loss. However, the loop bandwidth is limited to the lower bound, 4.5 Hz.

**Figure 23:**
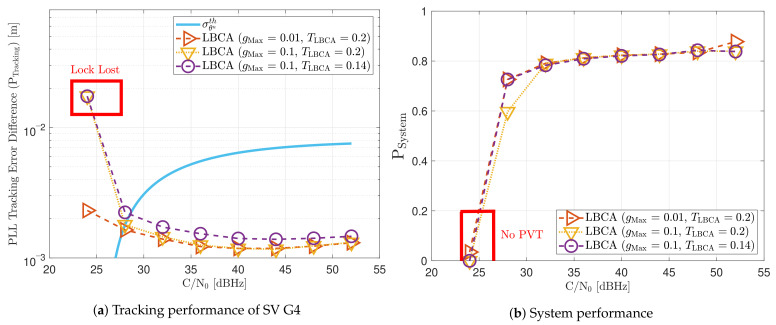
Static performance of loop-bandwidth control algorithm (LBCA) PLL at different *C*/*N*_0_ levels.

Figure 23 shows the static performance of the LBCA STL. The LBCA technique depends on the maximum value of the weighting function $g_{\mathrm{Max}}$ and the normalized dynamic threshold $T_{\mathrm{LBCA}}$ (see Figure 23a). As the FL technique, $g_{\mathrm{Max}}$ indicates the amount of oscillation of the loop bandwidth, and $T_{\mathrm{LBCA}}$ sets the sensitivity to signal dynamics. Figure 23a shows that a low $g_{\mathrm{Max}}$ (e.g., 0.01) can still track the signal at 24 dBHz. However, at a system level, the lock of at least 4 SV cannot be achieved and there is no PVT solution (see Figure 23b). An LBCA configuration with a higher $g_{\mathrm{Max}}$ makes the loop bandwidth update unstable at that *C*/*N*_0_ level, and even the lock of SV G4 is lost. It is also observed that a higher normalized dynamic threshold $T_{\mathrm{LBCA}}$ results in a worse tracking error difference.

**Figure 24:**
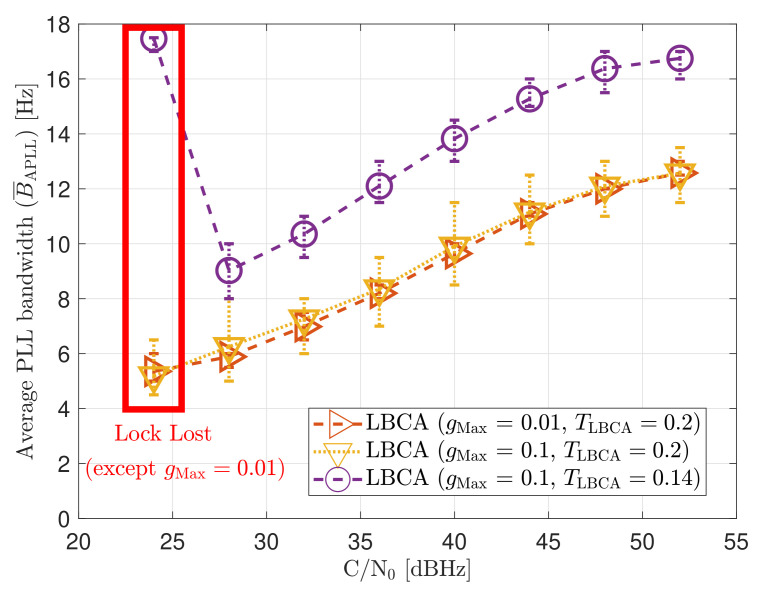
Average LBCA PLL loop bandwidth of SV G4 tracking in static scenario.

As expected, a lower $g_{\mathrm{Max}}$ leads to a smaller oscillation range of the estimated loop bandwidth (see Figure 24). Also, a decrease in $T_{\mathrm{LBCA}}$ increases the average loop bandwidth estimate.

**Figure 25:**
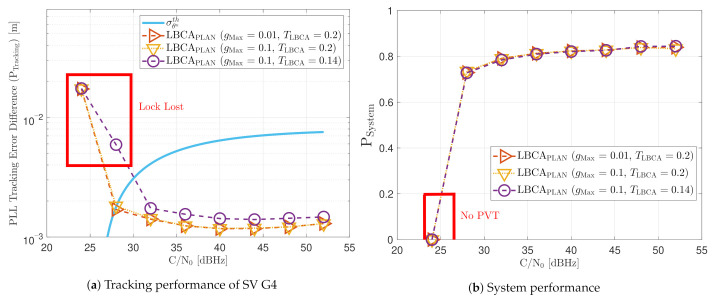
Static performance of LBCA PLL with PLAN interpolation at different *C*/*N*_0_ levels.

Figure 25 shows the tracking performance of the LBCA with PLAN interpolation STL. Due to approximation errors, the lock of SV G4 is not achieved at 24 dBHz even with a low $g_{\mathrm{Max}}$. These errors make the algorithm respond slower to achieve adequate loop bandwidth. Otherwise, the static tracking performance of this algorithm seems to be very similar to the LBCA technique.

**Figure 26:**
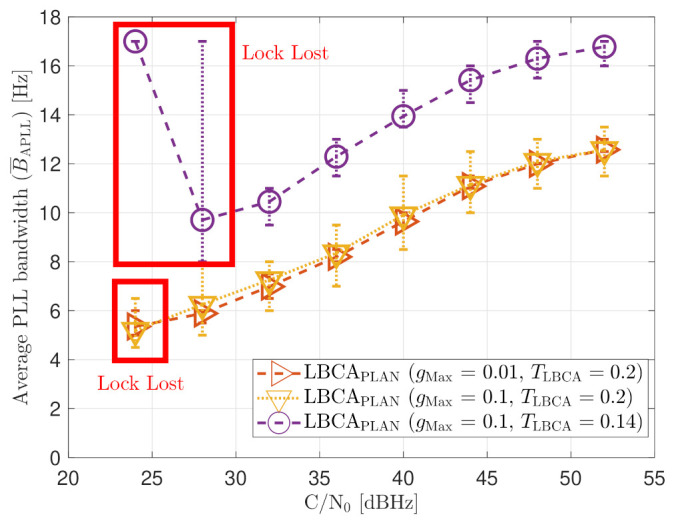
Average LBCA + PLAN PLL loop bandwidth of SV G4 tracking in static scenario.

Figure 26 reveals that the average loop bandwidth estimate of this technique is similar to the one without the piece-wise approximation.

### 6.2. Dynamic Scenario

**Figure 27:**
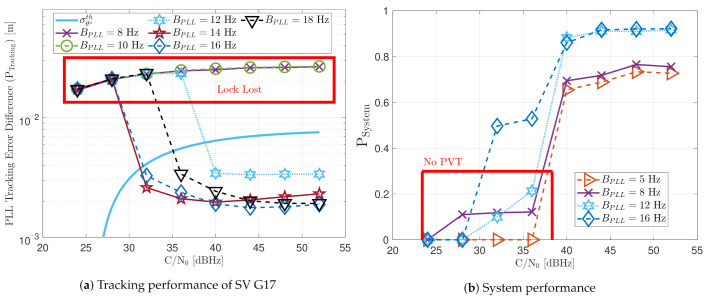
Dynamic performance of standard PLL at different *C*/*N*_0_ levels and loop bandwidths.

First, the performance of the standard tracking should be analyzed with different PLL loop bandwidths. Figure 27a shows that a loop bandwidth below 10 Hz loses the tracking lock of the SV G17 due to the large LOS dynamics at any *C*/*N*_0_ level. If the loop bandwidth is bigger than 10 Hz, the tracking can handle the LOS dynamics of SV G17. At lower *C*/*N*_0_ levels (e.g., 32 dBHz), a loop bandwidth of 14 Hz has the best performance, whereas, at a higher *C*/*N*_0_, a loop bandwidth between 16 Hz and 18 Hz is preferable. Figure 27b shows the system performance of the dynamic scenario. Loop bandwidths lower than 10 Hz achieve a PVT solution from 40 dBHz, mainly because it can track other SV with lower LOS dynamics. However, the system performance is still worse than higher bandwidths. Only a PLL loop bandwidth of 16 Hz can achieve a PVT down to 32 dBHz. At 28 dBHz, a loop bandwidth of 8 Hz has the best system performance, even without a PVT solution.

The following results present a detailed description of the adaptive tracking techniques’ capability to maintain the tracking lock against the dynamic scenario. Ideally, these techniques increase the loop bandwidth once signal dynamics are detected.

**Figure 28:**
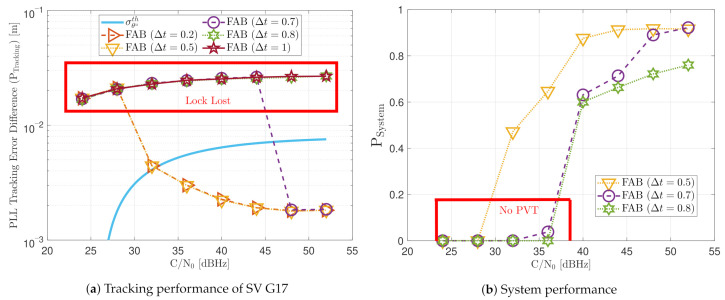
Dynamic performance of FAB at different *C*/*N*_0_ levels.

**Figure 29:**
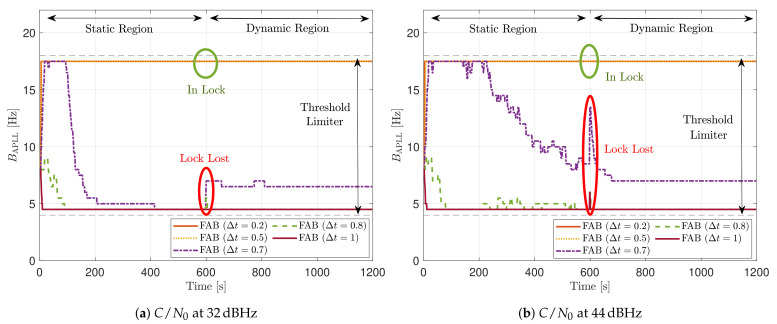
PLL loop bandwidth update with FAB of SV G17 tracking in dynamic scenario.

Figure 28 includes the tracking the tracking performance of SV 17 (Figure 28a) and the system performance (Figure 28b) of the FAB technique at different *C*/*N*_0_ levels. A lower filtered time Δt of the SSE estimator makes the algorithm react faster to dynamics. For instance, a Δt lower than 0.5 s presents better tracking performance than the other configurations. It also presents the best system performance. However, this configuration makes the algorithm too sensitive to dynamics, always having the maximum loop bandwidth possible (17.5 Hz) independent of the *C*/*N*_0_ level (see Figure 29). If Δt is greater than 0.7, the algorithm cannot track SV 17 at any *C*/*N*_0_ level. With this configuration, the loop bandwidth goes to the lower threshold 4.5 Hz at any *C*/*N*_0_ level. With Δt equal to 0.7 s, the estimated loop bandwidth achieves a value between the thresholds. Once dynamics are present, the FAB tries to increase the loop bandwidth to follow the signal dynamics. However, the algorithm does not succeed in maintaining the lock. A better solution should be a Δt between 0.5 s and 0.7 s.

**Figure 30:**
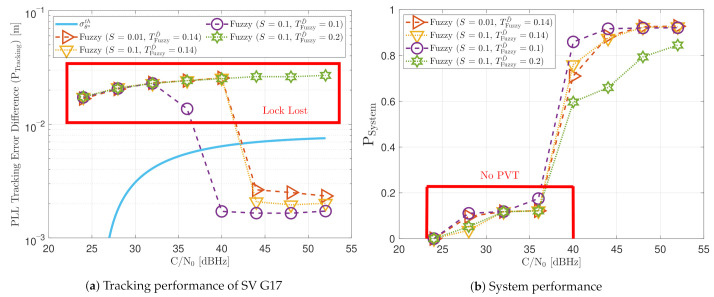
Dynamic performance of FL at different *C*/*N*_0_ levels.

Figure 30 shows the dynamic performance of the FL technique. A lower $T_{Fuzzy}^{\tilde{D}}$ presents better tracking and system performance. Figure 30a shows that a $T_{Fuzzy}^{\tilde{D}}$ equals to 0.1 achieves the lock of SV 17 down to 40 dBHz, whereas $T_{Fuzzy}^{\tilde{D}}=0.2$ do not achieve lock at any *C*/*N*_0_ level. Regarding the system performance, any configuration of the FL technique does not achieve a PVT solution up to 36 dBHz (see Figure 30b).

**Figure 31:**
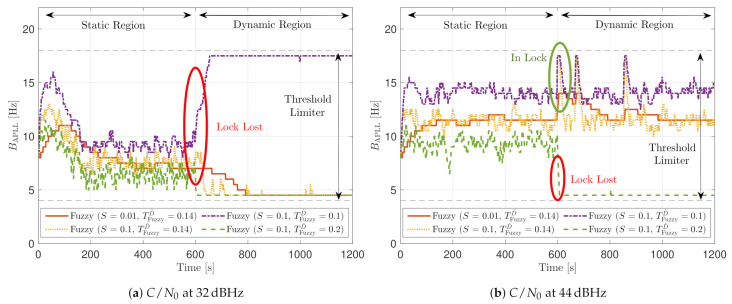
PLL loop bandwidth update with FL of SV G17 tracking in dynamic scenario.

Figure 31 shows how the PLL loop bandwidth adjusts during the dynamic scenario using the presented FL configurations. At 32 dBHz, all the configurations of the FL fail with tracking the signal. When the fuzzy technique with the lowest $T_{Fuzzy}^{\tilde{D}}$ loses lock, unlike the other configurations, the loop bandwidth goes to the maximum loop bandwidth threshold. This means that the loss of lock is considered as dynamics. This result is incorrect since the loop bandwidth should tend to zero to filter all the erroneous phase errors. At 44 dBHz, all the FL’s presented configurations can track the signal dynamics except with low sensitivity to dynamics, $T_{Fuzzy}^{\tilde{D}}=0.2$. Once this last configuration loses lock, the loop bandwidth goes to the lower loop bandwidth threshold, 4.5 Hz.

**Figure 32:**
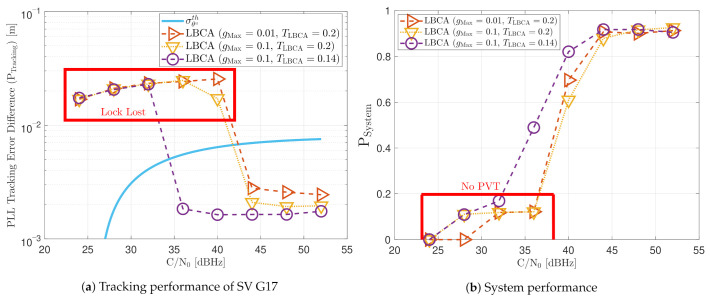
Dynamic performance of LBCA at different *C*/*N*_0_ levels.

**Figure 33:**
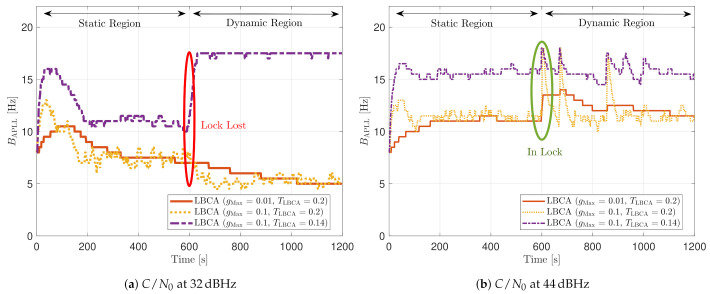
PLL loop bandwidth update with LBCA of SV G17 tracking in dynamic scenario.

Figure 32 and Figure 33 show that the LBCA technique presents a better dynamic performance with a lower dynamic threshold ($T_{\mathrm{LBCA}}=0.14$). A bigger scale factor $g_{\mathrm{Max}}$ improves the tracking performance of SV 17 (see Figure 32a) because of the bigger loop bandwidth update. However, a bigger update can lead also to instabilities and worse performance in the presence of lower LOS dynamics. Figure 32b shows that, at $T_{\mathrm{LBCA}}=0.2$, a lower scale factor can achieve better system performance at some *C*/*N*_0_ levels (e.g., 40 dBHz).

At 32 dBHz, the LBCA technique cannot achieve lock at any configurations (see Figure 33a). The same as in the FL technique happens when the algorithm is too sensitive to dynamics (e.g., $T_{\mathrm{LBCA}}=0.14$) that it goes erroneously to the highest loop bandwidth when the lock is lost. At 44 dBHz, all the configurations can track SV 17 during the dynamic scenario. Figure 33b presents the effects of the dynamic threshold and the scale factor. A bigger dynamic threshold leads to bigger bias of the estimated loop bandwidth. This is also observed in Figure 24. Moreover, a bigger scale factor results in a noisier loop bandwidth estimate, but with a better capability to react faster.

**Figure 34:**
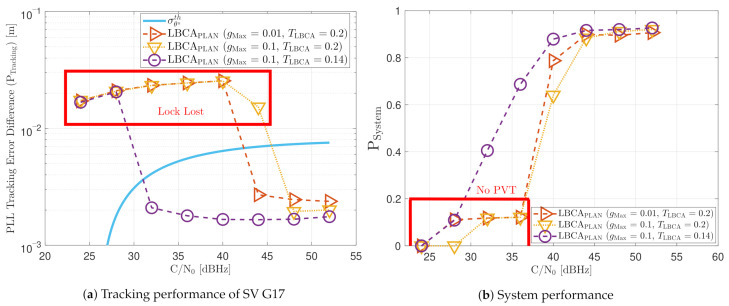
Dynamic performance of LBCA with PLAN interpolation at different *C*/*N*_0_ levels.

The LBCA with PLAN interpolation presents a similar dynamic performance as the LBCA at high *C*/*N*_0_ levels (see Figure 34). At lower *C*/*N*_0_, the dynamic performance using a scale factor of $g_{\mathrm{Max}}=0.1$ and a normalized dynamics threshold $T_{LBCA}=0.14$ stands out above the rest of the configurations. This configuration maintains the lock even at 32 dBHz. In addition, the configuration with $g_{\mathrm{Max}}=0.1$ and $T_{LBCA}=0.2$ has poor tracking performance at 44 dBHz. However, the system performance is similar to the other configurations at that *C*/*N*_0_ level.

**Figure 35:**
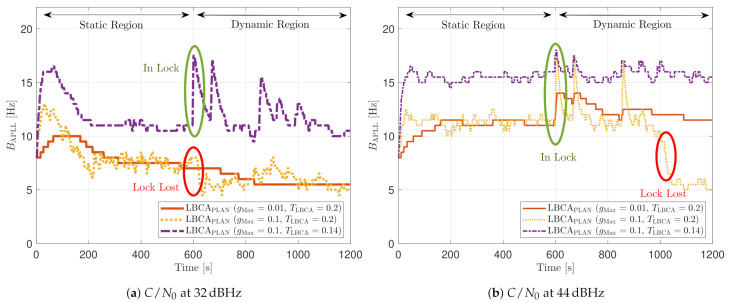
PLL loop bandwidth update with LBCA with PLAN interpolation of SV G17 tracking in dynamic scenario

The LBCA + PLAN technique with S=0.1 and $T_{LBCA}=0.14$ is the unique adaptive tracking technique that can still track the signal at 32 dBHz in the presence of high LOS jerk dynamics. Once dynamics are present, the estimated loop bandwidth goes from 11 Hz to 17.5 Hz to follow the phase error dynamics (see Figure 35a). After that, the loop bandwidth goes back to 11 Hz and repeats when new dynamics are detected.

The LBCA + PLAN technique with S=0.1 and $T_{LBCA}=0.2$ loses lock at time t=1000 s (see Figure 35b). This explains the poor tracking performance of this configuration at that *C*/*N*_0_ level (see Figure 34a). The high scale-factor causes unstable loop bandwidth estimates. It can happen that, once dynamics are present, the loop bandwidth estimate has such a low value that it is not able to respond to signal dynamics and loses lock.

### 6.3. Total System Performance

This section summarizes the previous sections’ obtained results, taking a single quality factor that determines the adaptive tracking techniques’ system performance in a static and a dynamic scenario. The average system performance ${\bar{P}}_{\mathrm{System}}$ (see Equation (55)) for both the static and dynamic scenario is calculated and displayed in Table 6. The labels-column in this table is used for further analysis in a subsequent section. Furthermore, for each tracking technique, the best system performance is marked in green, whereas the worst one is in red.

### 6.4. Performance vs. Complexity Comparison

**Figure 36:**
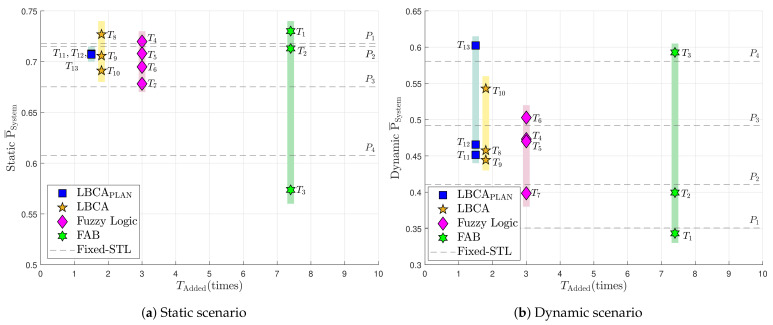
System performance vs. added time complexity comparison.

Figure 36 contains the performance and complexity comparison of the implemented adaptive tracking techniques for a third-order PLL in a static (Figure 36a) and dynamic scenario (Figure 36b). These graphs combine the data of Table 2 and Table 6.

In the static scenario, the standard tracking loop has the best average system performance with a PLL loop bandwidth of 5 Hz ($P_{1}$), whereas the worst performance is performed with 16 Hz ($P_{4}$). However, the opposite occurs in the dynamic scenario. A greater loop bandwidth improves the average system performance. This shows the big trade-off that exists in standard tracking loops between the loop bandwidth and the type of scenario.

The adaptive tracking algorithms should minimize this trade-off, having a good performance in both scenarios. The FAB technique with a high decay time Δt (e.g., 0.8 s, $T_{1}$) results in best performance in static scenario, but poor in dynamic scenario. A lower decay time (e.g., 0.5 s, $T_{3}$) have the opposite behavior—poor in static and good in dynamic. There is no minimization of the mentioned trade-off. The FAB technique presents poor results, while having high complexity.

The FL technique presents good system performance in a static scenario with $T_{\mathrm{Fuzzy}}^{\tilde{D}}=0.14$ ($T_{4},T_{5}$). A lower ($T_{6}$) or a higher ($T_{7}$) fuzzy threshold decreases the performance. In a dynamic scenario, a lower fuzzy ($T_{6}$) threshold achieves better performance. $T_{6}$, $T_{5}$ and $T_{4}$ show an improvement over the standard PLL. However, the complexity is still significant.

The LBCA and LBCA with PLAN interpolation achieve good system performance, while having a low complexity. In the LBCA technique, a lower scale factor $g_{\mathrm{Max}}$ presents better performance in the static and the dynamic scenario with the same normalized dynamics threshold ($T_{\mathrm{LBCA}}=0.2$, $T_{8}$ and $T_{9}$). Furthermore, a lower $T_{\mathrm{LBCA}}$ deteriorates the static system performance, but improves the dynamic one. In the LBCA with PLAN interpolation, the presented configurations ($T_{11},T_{12},T_{13}$) have the same good static system performance. The dynamic performance of $T_{13}$ stands out above all the adaptive tracking techniques. To conclude, this technique presents the best performance, maintaining the lowest complexity.

## 7. Discussion

The previous section collects all the obtained results to evaluate the FAB, FL, LBCA, and LBCA + PLAN techniques. The adaptive tracking techniques are evaluated in a static and a dynamic scenario at different *C*/*N*_0_ levels. For each scenario, one specific tracking channel (SV G4 in static, SV G17 in dynamic) is analyzed to understand how the presented algorithms work using different configurations. In addition, the metric for the system performance (see Equation (55)) is measured for each adaptive tracking technique and compared to its complexity (see Figure 36).

**Figure 37:**
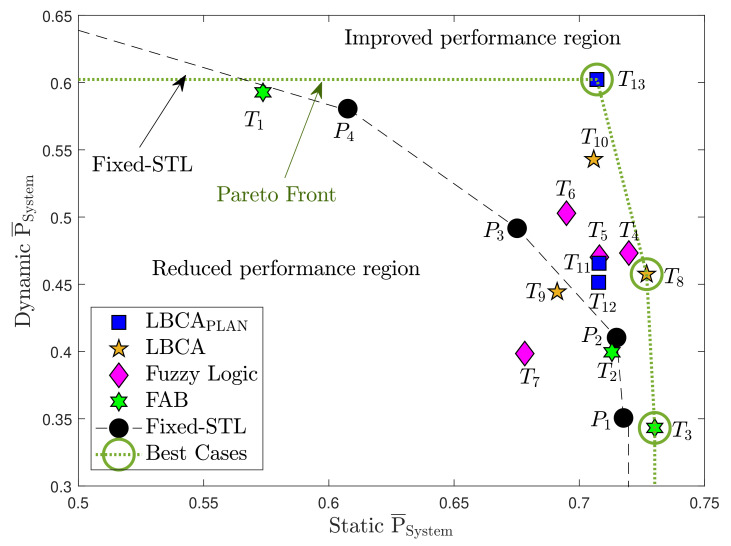
System performance comparison between dynamic and static scenario.

Figure 37 compares the measured static system performance with the dynamic system performance of the adaptive tracking techniques with different configurations. This Figure is based on Table 6 and synthesizes all the previous sections’ results into a single graph. The performance of the standard PLL at different loop bandwidths is also included. The dashed black line is an interpolation of the standard tracking results. Above this line, the values present a better system performance than the standard PLL, whereas results located below this line indicate a worse system performance. The STL clearly demonstrates the loop-bandwidth trade-off between static and dynamic scenarios The best-performing adaptive tracking technique should have the highest average system performance in static and dynamic scenarios.

The FAB technique ($T_{1},T_{2},T_{3}$) has a similar performance as the standard PLL while having the highest complexity. Therefore, although not all the possible configurations have been tested, it seems that this technique does not bring any improvement. A better choice of the decay time Δt can improve the performance of this algorithm. The FL technique presents better performance than the standard tracking except for one configuration, $T_{7}$. A fuzzy threshold $T_{\mathrm{Fuzzy}}^{\tilde{D}}$ of 0.2 makes the algorithm too sensitive to noise, worsening the dynamic performance (see Figure 30b). Also, the high scale factor (S=0.1) results in a more unstable tracking, leading to a worse static performance at low *C*/*N*_0_ (see Figure 21b). The other configurations ($T_{4},T_{5},T_{6}$) are above the standard PLL’s performance. A lower fuzzy threshold $T_{\mathrm{Fuzzy}}^{\tilde{D}}$ than configuration $T_{7}$ gains dynamic and static performance ($T_{5},T_{4}$). If $T_{\mathrm{Fuzzy}}^{\tilde{D}}$ is even lower ($T_{6}$), the dynamic performance improves with the trade-off of losing some static performance. The LBCA technique also achieves a good performance except for $T_{9}$. A large scale factor ($g_{\mathrm{Max}}=0.1$) with a high normalized threshold dynamics $T_{\mathrm{LBCA}}$, as in the FL technique, can make the tracking unstable. A lower scale factor ($T_{8}$) significantly improves the static performance and slightly the dynamic performance, whereas a low threshold dynamics ($T_{10}$) significantly improves the dynamic performance and slightly the static one. Finally, the LBCA with PLAN technique presents better performance than standard PLL in all the presented configurations ($T_{11},T_{12},T_{13}$). These three configurations have the same static performance, and the dynamic performance is improved significantly when the normalized threshold dynamics is equal to the optimal normalized dynamics ($T_{\mathrm{LBCA}}=1/7$).

The green dotted line in the top right of the graph is the Pareto front [49,50,51]. It isolates a set of optimal values to facilitate the trade-off between static and dynamic performance. All other values are sub-optimal in the Pareto sense. There are three values on the Pareto front: $T_{3}$, $T_{8}$ and $T_{13}$. However, $T_{3}$ (FAB) exhibits faulty behavior as it always adapts to the minimum loop bandwidth of 4.5 Hz, and cannot be used for reliable tracking. Therefore, it is excluded. The remaining correct performance-optimal adaptive tracking techniques are LBCA with PLAN using S=0.1 and $T_{\mathrm{LBCA}}=0.14$ ($T_{13}$), and LBCA with S=0.01 and $T_{\mathrm{LBCA}}=0.2$ ($T_{8}$). The optimization demonstrates the superiority of LBCA based adaptive algorithms with the correct tuning.

## 8. Conclusions

This paper presents a method to compare the performance and complexity of different state-of-the-art adaptive loop-bandwidth tracking techniques. First, the theoretical background of an STL is presented. Second, the FAB, the FL, and the LBCA adaptive tracking techniques are described. Third, the metrics used to evaluate the adaptive tracking techniques are analyzed. Furthermore, each adaptive tracking technique’s complexity is measured in terms of the required number of operations as well as the time complexity. Fourth, the algorithms are implemented on the GOOSE receiver platform with simulated static and dynamic scenarios. The dynamic scenarios represent harsh vehicular movement.

The results show each algorithm’s tracking and system performance in a static and a dynamic simulated scenario. The FAB presents insignificant improvements compared to STLs, has poor system performance and is the most complex adaptive algorithm. The FL is conceptually simple and shows—when tuned correctly– significant improvement to STLs. It is an algorithm with much potential. The LBCA and LBCA + PLAN techniques have superior system performance with the least amount of complexity. These algorithms–depending on the configurations–are also determined optimal in the static to dynamic trade-off, illustrating a clear advantage.

A broader statistical evaluation to test the adaptive tracking techniques with a wider variety of configurations, *C*/*N*_0_ levels, and scenarios is proposed for future work. It can additionally include multi-path and non-line-of-sight (NLOS) effects. This would improve the generalization and applicability of the adaptive tracking algorithms, and make these less dependent on the chosen scenarios. However, this paper presents an initial investigation and includes a framework to evaluate the adaptive techniques. The adaptive techniques in this paper are only evaluated for the PLL. However, a tracking channel also has a DLL and FLL. Therefore, future work to incorporate adaptive techniques to these loops are also proposed. The results for the LBCA and FL have shown that these algorithms’ performances are sensitive to their configurations. Therefore, further research to optimize the configurations are suggested. Furthermore, a vector-based LBCA approach which considers multiple GNSS SVs simultaneously is also suggested. Lastly, an extension of the adaptive loop-bandwidth algorithm can be done by also adapting the integration time.

This paper encloses an initial study of robust tracking techniques with the primary objective of developing a method that achieves the best performance while maintaining low complexity. The LBCA with the PLAN interpolation is currently the best candidate among the other techniques.

## Figures and Tables

**Table 1:** Structure of the fuzzy Matrix $W_{3\times 3}^{\mathrm{fuzzy}}$.

		$\tilde{D}$		
		ZO	PS	PL
**$\tilde{N}$**	ZO	0	>0	>0
	PS	<0	0	>0
	PL	<0	<0	0

**Table 2:** Complexity of the adaptive loop-bandwidth tracking techniques based on the number of operations.

Tracking Technique	Sub-Module	Number of Operations:			
		Additions	Multiplications	Divisions	Other
FAB	Dynamic Stress Estimator	4	6	1	-
	Three-Sigma FAB	1	11	2	$\sqrt[{7}]{\cdot }$
	Gradient Descent	3	1	1	-
	Total	8	18	4	1
FL	Dynamic/Noise Estimator	4	4	2	-
	FL Algorithm	13	25	0	-
	Total	17	29	2	0
LBCA	Dynamic/Noise Estimator	2	1	1	-
	LBCA algorithm	4	5	1	exp
	Total	6	6	2	1
LBCA + PLAN	Dynamic/Noise Estimator	2	1	1	-
	LBCA algorithm	4	6	0	-
	Total	6	7	1	0

**Table 3:** Time complexity of the adaptive loop-bandwidth tracking techniques, $3\times 10^{8}$ iterations.

Tracking Technique	Total Time Complexity $T_{C}$ (s)	Iteration Time Complexity $T_{\mathrm{Iter}}$ (ns)	Added Time Complexity $T_{\mathrm{Added}}$ (Times)
Standard	18.1	60.3	1
FAB	133.7	445.8	7.4×
FL	55.0	183.5	3.0×
LBCA	31.9	106.4	1.8×
LBCA + PLAN	27.8	92.8	1.5×

**Table 4:** Initial configuration of the DLL, FLL and PLL for each tracking channel.

	DLL	FLL	PLL
Order	2	2	3
*B* [Hz]	0.1	15	8
Discriminator	CELP	Diff Atan	Atan

**Table 5:** Selected values of the fuzzy Matrix $W_{3\times 3}^{\mathrm{fuzzy}}$.

		$\tilde{D}$		
		ZO	PS	PL
**$\tilde{N}$**	ZO	0	0.5	0.75
	PS	−0.25	0	0.5
	PL	−0.5	−0.25	0

**Table 6:** System performance of adaptive tracking techniques.

Tracking Technique	Configuration	Label	${\bar{P}}_{\mathrm{System}}$ Static	${\bar{P}}_{\mathrm{System}}$ Dynamic
Standard PLL	$B_{\mathrm{PLL}}=5\mathrm{Hz}$	$P_{1}$	0.718	0.351
	$B_{\mathrm{PLL}}=8\mathrm{Hz}$	$P_{2}$	0.715	0.410
	$B_{\mathrm{PLL}}=12\mathrm{Hz}$	$P_{3}$	0.675	0.492
	$B_{\mathrm{PLL}}=16\mathrm{Hz}$	$P_{4}$	0.608	0.581
FAB	Δt= 0.8 s	$T_{1}$	0.730	0.343
	Δt= 0.7 s	$T_{2}$	0.713	0.399
	Δt= 0.5 s	$T_{3}$	0.574	0.593
FL	S=0.01, $T_{\mathrm{Fuzzy}}^{\tilde{D}}=0.14$	$T_{4}$	0.720	0.473
	S=0.1, $T_{\mathrm{Fuzzy}}^{\tilde{D}}=0.14$	$T_{5}$	0.708	0.470
	S=0.1, $T_{\mathrm{Fuzzy}}^{\tilde{D}}=0.1$	$T_{6}$	0.695	0.503
	S=0.1, $T_{\mathrm{Fuzzy}}^{\tilde{D}}=0.2$	$T_{7}$	0.678	0.398
LBCA	S=0.01, $T_{\mathrm{LBCA}}=0.2$	$T_{8}$	0.727	0.458
	S=0.1, $T_{\mathrm{LBCA}}=0.2$	$T_{9}$	0.691	0.444
	S=0.1, $T_{\mathrm{LBCA}}=0.14$	$T_{10}$	0.706	0.543
LBCA + PLAN	S=0.01, $T_{\mathrm{LBCA}}=0.2$	$T_{11}$	0.708	0.466
	S=0.1, $T_{\mathrm{LBCA}}=0.2$	$T_{12}$	0.708	0.451
	S=0.1, $T_{\mathrm{LBCA}}=0.14$	$T_{13}$	0.707	0.602

## Data Availability

Publicly available datasets were analyzed in this study. This data can be found here: https://owncloud.fraunhofer.de/index.php/s/LGoWPVtV5xbQ9mB.

## Abbreviations

The following abbreviations are used in this manuscript:

ADC	analog-to-digital converter
BET	backward Euler transform
$C/N_{0}$	carrier-to-noise density ratio
COG	center of gravity
CRB	Cramér-Rao bound
DLL	delay locked loop
FAB	fast adaptive bandwidth
FAP	FLL-assisted-PLL
FL	fuzzy logic
FLL	frequency locked loop
FMM	fuzzy mean method
FPGA	field-programmable gate array
GNSS	global navigation satellite system
GOOSE	GNSS-Receiver with Open Software Interface
GPS	Global Positioning System
IAD	integration and dump
IIR	infinite impulse response
IQ	in-phase and quadrature-phase
KF	Kalman filtering
LBCA	loop-bandwidth control algorithm
LOS	line-of-sight
NCO	numerically controlled oscillator
NLOS	non-line-of-sight
OGRP	open GNSS receiver protocol
PAD	PLL-assisted-DLL
PCIe	peripheral component interconnect express
PLAN	piecewise linear approximation of nonlinearities
PLI	phase-lock indicator
PLL	phase locked loop
PVT	position, velocity, and time
RFCS	radio-frequency constellation simulator
RFFE	radio-frequency front-end
SSE	steady state error
SSM	state space model
STL	scalar tracking loop
SV	satellite vehicle
TCXO	temperature-compensated crystal oscillator
ToA	time of arrival
VT	vector tracking
